# Three-Layered Complex Interactions among Capsidless (+)ssRNA Yadokariviruses, dsRNA Viruses, and a Fungus

**DOI:** 10.1128/mbio.01685-22

**Published:** 2022-08-30

**Authors:** Yukiyo Sato, Sakae Hisano, Carlos José López-Herrera, Hideki Kondo, Nobuhiro Suzuki

**Affiliations:** a Agrivirology Laboratory, Institute of Plant Science and Resources, Okayama University, Kurashiki, Okayama, Japan; b Instituto de Agricultura Sostenible C.S.I.C., Alameda del Obispo, Córdoba, Spain; Indiana University Bloomington

**Keywords:** virus-virus interaction, RNA viruses, capsidless, virus macroevolution, fungal viruses, plant-pathogenic fungi, mutualism and parasitism, multilayered interaction

## Abstract

We have previously discovered a virus neo-lifestyle exhibited by a capsidless positive-sense (+), single-stranded (ss) RNA virus YkV1 (family *Yadokariviridae*) and an unrelated double-stranded (ds) RNA virus YnV1 (proposed family “*Yadonushiviridae*”) in a phytopathogenic ascomycete, *Rosellinia necatrix*. YkV1 has been proposed to replicate in the capsid provided by YnV1 as if it were a dsRNA virus and enhance YnV1 replication in return. Recently, viruses related to YkV1 (yadokariviruses) have been isolated from diverse ascomycetous fungi. However, it remains obscure whether such viruses generally show the YkV1-like lifestyle. Here, we identified partner viruses for three distinct yadokariviruses, YkV3, YkV4a, and YkV4b, isolated from *R. necatrix* that were coinfected with multiple dsRNA viruses phylogenetically distantly related to YnV1. We first established transformants of *R. necatrix* carrying single yadokarivirus cDNAs and fused them with infectants by single partner candidate dsRNA viruses. Consequently, YkV3 and YkV4s replicated only in the presence of RnMBV3 (family *Megabirnaviridae*) and RnMTV1 (proposed family “*Megatotiviridae*”), respectively. The partners were mutually interchangeable between the two YkV4 strains and three RnMTV1 strains but not between other combinations involving YkV1 or YkV3. In contrast to YkV1 enhancing YnV1 accumulation, YkV4s reduced RnMTV1 accumulation to different degrees according to strains. Interestingly, YkV4 rescued the host *R. necatrix* from impaired growth induced by RnMTV1. YkV3 exerted no apparent effect on its partner (RnMBV3) or host fungus. Overall, we revealed that while yadokariviruses generally require partner dsRNA viruses for replication, each yadokarivirus partners with a different dsRNA virus species in the three diverse families and shows a distinct symbiotic relation in a fungus.

## INTRODUCTION

RNA viruses, a major part of the global eukaryotic virome (1–6), generally package the genomes in their own capsid protein (CP) and replicate using their own RNA-directed RNA polymerase (RdRP), which is the hallmark of all RNA viruses. Positive-sense (+), single-stranded (ss) RNA viruses usually utilize host membrane-derived compartments or spherules to replicate their genomes, while negative-sense (–) ssRNA and double-stranded (ds) RNA viruses encase their own RdRP in the virions or particle intermediates to synthesize viral RNAs (7). Viruses often coinfect single host organisms, and this is particularly true for viruses of filamentous fungi, where several types of virus/virus interactions occur in single hosts (8). Among them is a recently discovered unusual mutualistic interaction between two phylogenetically distinct viruses in a phytopathogenic filamentous ascomycetous fungus, *Rosellinia necatrix*. Therein, a capsidless, (+)ssRNA virus termed yadokari virus 1 (YkV1) borrows the capsid of a dsRNA virus termed yadonushi virus (YnV1) and enhances YnV1 replication in return (9, 10). The yado-kari (“room borrower” in Japanese) nature is defined as being hosted or heteroencapsidated by a dsRNA virus, while the yado-nushi (“room owner” in Japanese) nature is defined as hosting or *trans*-encapsidating yadokariviruses (11). YkV1 RdRP shows low phylogenetic relationships with RdRPs of animal-infecting caliciviruses with (+)ssRNA genomes, members of the extended picorna-like supergroup (phylum *Pisviricota*) (1), exemplified by sapo- and noroviruses. YkV1 possesses a single open reading frame (ORF) encoding an RdRP domain and a 2A-like self-cleavage peptide motif (–GDVEKNPG↓P–) that triggers ribosome skipping (12). YnV1 is a full-fledged independent dsRNA virus and phylogenetically distantly related to dsRNA toti-like viruses or members of the order *Ghabrivirales*. The YnV1 genome has two ORFs encoding CP and RdRP that are assumed to be expressed as a CP-RdRP fusion protein via −1 frame-shifting (9, 13). YkV1 RNA and its RdRP have been shown to be *trans*-encapsidated by YnV1 CP, and YkV1 RdRP is necessary for the YkV1 replication (14). Based on these observations, YkV1 has been hypothesized to replicate in the YnV1 heterocapsid as the replication site as if it were a dsRNA virus (9, 10, 14).

**FIG 1:**
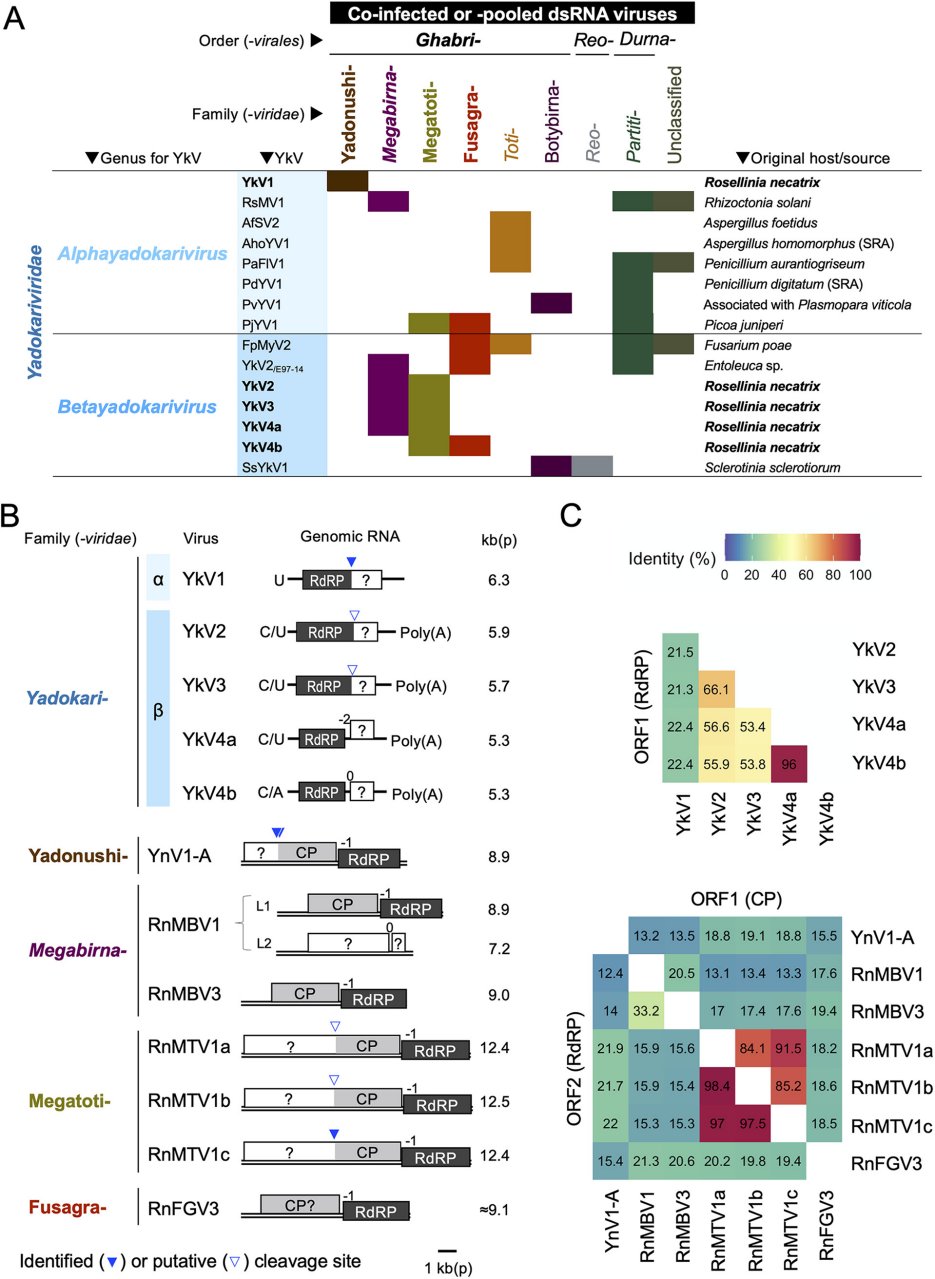
Yadokariviruses and candidate partner dsRNA viruses. (A) Combinations of yadokariviruses (YkV) and dsRNA viruses found in the same fungal isolates or metagenomic pools. See Table S1 for references. The copresent yadokariviruses and dsRNA viruses are filled. The dsRNA viruses are shown by the family-level taxa to which they belong. The officially established taxa are italicized, while previously proposed ones were not. The parenthesized SRA means that corresponding viruses were detected from publicly available short-read datasets in NCBI Sequence Read Archive (SRA). (B) Genome organization of the yadokariviruses and their candidate partner viruses used in this study. Yadokariviruses in the genus *Alphayadokarivirus* or *Betayadokarivirus* are indicated with α or β, respectively. Accession numbers for the respective genome sequences are listed in Table S3. The blue triangles indicate experimentally determined (filled) or putative (open) cleavage sites for polyproteins. (C) Percent identity matrix of putative viral proteins based on global amino acid sequence alignment. The top matrix shows the comparison among proteins encoded by ORF1 (RdRP) of the yadokariviruses. The bottom matrixes show the comparison among proteins encoded by ORF1 (CP) or ORF2 (RdRP) of the dsRNA viruses (top right and bottom left, respectively).

Recent virus-hunting studies have found many viruses that have close phylogenetic relationships to YkV1 and lack putative capsid proteins, from diverse ascomycetous fungi or undetermined hosts (3, 15–22) (Fig. 1A and Table S1 in the supplemental material). To accommodate these viruses, we have proposed to create an order *Yadokarivirales* and a family *Yadokariviridae* in the phylum *Pisuviricota* (picornavirus supergroup) to the International Committee on Taxonomy of Viruses ICTV; https://ictv.global/taxonomy/taxondetails?taxnode_id=202112467, ICTV approved. These members can further be divided into two genera *Alphayadokarivirus* and *Betayadokarivirus*, based on the distinct phylogenetic clades. Examples of alphayadokariviruses include YkV1 and Aspergillus foetidus slow virus 2 (AfSV2) (18). Examples of betayadokariviruses include yado-kari virus 2, 3, and 4 (YkV2, YkV3, and YkV4) from *R. necatrix*. YkV4 differs from YkV1 in genome organization with two-ORF structures (15). Interestingly, YkV2, YkV3, and YkV4 were found with no YnV1-like dsRNA viruses, instead, with dsRNA viruses very distantly related to YnV1, i.e., members of the existing family *Megabirnaviridae* and the proposed families “*Fusagraviridae*” and “*Megatotiviridae*,” all being within the order *Ghabrivirales* (Fig. 1A and Fig. S1). The other yadokariviruses have also been found with diverse dsRNA viruses (Fig. 1A). However, it is unknown whether these yadokariviruses are commonly dependent on partner dsRNA viruses and, if yes, which dsRNA viruses they partner and how they affect partner viruses and hosts.

10.1128/mbio.01685-22.1 FIG S1 Phylogenetic relationships of selected viruses in the order *Ghabrivirales*. The phylogenetic relationships were analyzed based on the amino acid sequences of RdRPs listed in Table S2. Several representative viruses and the viruses that were reported to be coinfected with yadokariviruses (Fig. 1A) were involved in the analysis. The viruses assumed to be coinfected with alphayadokariviruses and betayadokariviruses are indicated with red stars or blue hexagrams, respectively. The values flanking the nodes indicate bootstrap probability in 1000 iterations. Download FIG S1, PDF file, 0.04 MB. Copyright © 2022 Sato et al. 2022 Sato et al. https://creativecommons.org/licenses/by/4.0/ This content is distributed under the terms of the Creative Commons Attribution 4.0 International license.

10.1128/mbio.01685-22.6 TABLE S1 Viruses in the family *Yadokariviridae*. Download Table S1, DOCX file, 0.04 MB. Copyright © 2022 Sato et al. 2022 Sato et al. https://creativecommons.org/licenses/by/4.0/ This content is distributed under the terms of the Creative Commons Attribution 4.0 International license.

10.1128/mbio.01685-22.8 TABLE S3 Accession numbers for viral sequences used in this study. Download Table S3, DOCX file, 0.03 MB. Copyright © 2022 Sato et al. 2022 Sato et al. https://creativecommons.org/licenses/by/4.0/ This content is distributed under the terms of the Creative Commons Attribution 4.0 International license.

In this study, we identified partner dsRNA viruses for the betayadokariviruses YkV3, YkV4a, and YkV4b and performed partner swapping assays among them and YkV1. Our results suggest that these yadokariviruses commonly have the yadokari nature but each partners with different dsRNA viruses in diverse families. Furthermore, the effects of these yadokariviruses on their partner viruses and host *R. necatrix* were diverse, from mutualistic, commensal, to parasitic. We will provide the catalog of three-layered symbiotic relationships among yadokariviruses, diverse dsRNA viruses, and a host fungus.

## RESULTS

### Novel dsRNA virus partner candidates for the yadokariviruses.

A total of five yadokariviruses (YkV1, YkV2, YkV3, YkV4a, and YkV4b) were reported from three *R. necatrix* isolates, namely, Japanese W1032 and Spanish Rn95-16 and Rn454, that were coinfected with diverse RNA viruses (9, 15) (Table 1 and Fig. 1B). Only YkV1’s dsRNA virus partner was earlier identified as YnV1 with three variants (A to C); both viruses were found to be from W1032. In the Spanish fungal strain Rn454, a dsRNA megabirnavirus (Rosellinia necatrix megatotivirus 3 [RnMBV3]) and three betayadokariviruses (YkV2, YkV3, and YkV4a) have been identified. In addition, we identified a novel megatotivirus termed Rosellinia necatrix megatotivirus 1 (RnMTV1, strain a) from the Rn454 in this study. The Spanish fungal strain Rn95-16 was coinfected by a second RnMTV1 isolate (named strain b) and a fusagravirus, *Rosellinia necatrix* fusagravirus 3 (RnFGV3), in addition to another YkV4 isolate (YkV4b). YkV4a and YkV4b represent two different strains of YkV4, which share 96.0% global amino acid sequence identity in RdRP (Fig. 1C). Another megatotivirus strain (the third RnMTV1 strain, c), a candidate partner dsRNA virus (see below), was found in a yadokarivirus-absent Spanish *R. necatrix* strain Rn430, along with a dsRNA partitivirus and a (+)ssRNA hypovirus (Table 1). The three megatotivirus strains (RnMTV1a, b, c) shared 84.1% to 91.5% and 97.0% to 98.5% amino acid sequence identities in ORF1-encoded protein and RdRP, respectively (Fig. 1C). The yadokarivirus-partner candidate viruses, YnV1 (a yadonushivirus), RnMBV3 (a megabirnavirus), RnMTV1a/b/c (three strains of a megatotivrius), and RnFGV3 (a fusagravirus) commonly have a similar two-ORF or totivirus-like genome architecture (Fig. 1B). Moreover, they or their relatives form spherical particles 40 to 50 nm in diameter (9, 23–25) (see below). In contrast, their putative RdRP and CP (polyprotein containing CP) share little sequence identity among different viral species (12.4% to 22.0% for RdRP and 13.1% to 19.4% for CP) (Fig. 1C).

**TABLE 1:** Virus and fungal strains used in this study

Fungal strain	Virus strains harbored	Virus strain abbreviation	Accession	Reference
*R. necatrix* W97 (Japanese)	Virus-free standard strain			29
*R. necatrix* W1032 (Japanese)	Yadonushi virus 1-A	YnV1-A	LC061478	9
	Yadonushi virus 1-B	YnV1-B	LC006254	10
	Yadonushi virus 1-C	YnV1-C	LC006256	10
	Yadokari virus 1	YkV1	LC006253	9
*R. necatrix* W779 (Japanese)	Rosellinia necatrix megabirnavirus 1	RnMBV1	AB512282, AB512283	30
*R. necatrix* Rn454 (Spanish)^*a*^	Rosellinia necatrix megabirnavirus 3	RnMBV3	LC333756	15
	Rosellinia necatrix megatotivirus 1a	RnMTV1a	LC650957	This study
	Yadokari virus 2	YkV2	LC333755	15
	Yadokari virus 3	YkV3	LC333757	15
	Yadokari virus 4a	YkV4a	LC333754	15
*R. necatrix* Rn95-16 (Spanish)^*a*^	Rosellinia necatrix fusagravirus 3	RnFGV3	LC333739	15
	Rosellinia necatrix megatotivirus 1b	RnMTV1b	LC333740	15
	Yadokari virus 4b	YkV4b	LC333741	15
*R. necatrix* Rn430 (Spanish)	Rosellinia necatrix megatotivirus 1c	RnMTV1c	LC333746	15
	Rosellinia necatrix hypovirus 2	RnHV2	LC333745	15
	Rosellinia necatrix partitivirus 9	RnPV9	LC333747, LC333748	15

a Several unlisted viral sequences (hypovirus-like short contigs LC333749 to LC333752) in Rn454; fusari-like short contigs [LC333742 to LC333744] and Rosellinia necatrix fusagravirus 2 [RnFGV2] LC333738 in Rn95-16; and RnFGV3 [LC333739] in Rn430 were previously reported (15), which were not detected in this study (Fig. S2).

### Identification of intrinsic partner dsRNA viruses for YkV3 and YkV4.

**FIG 2:**
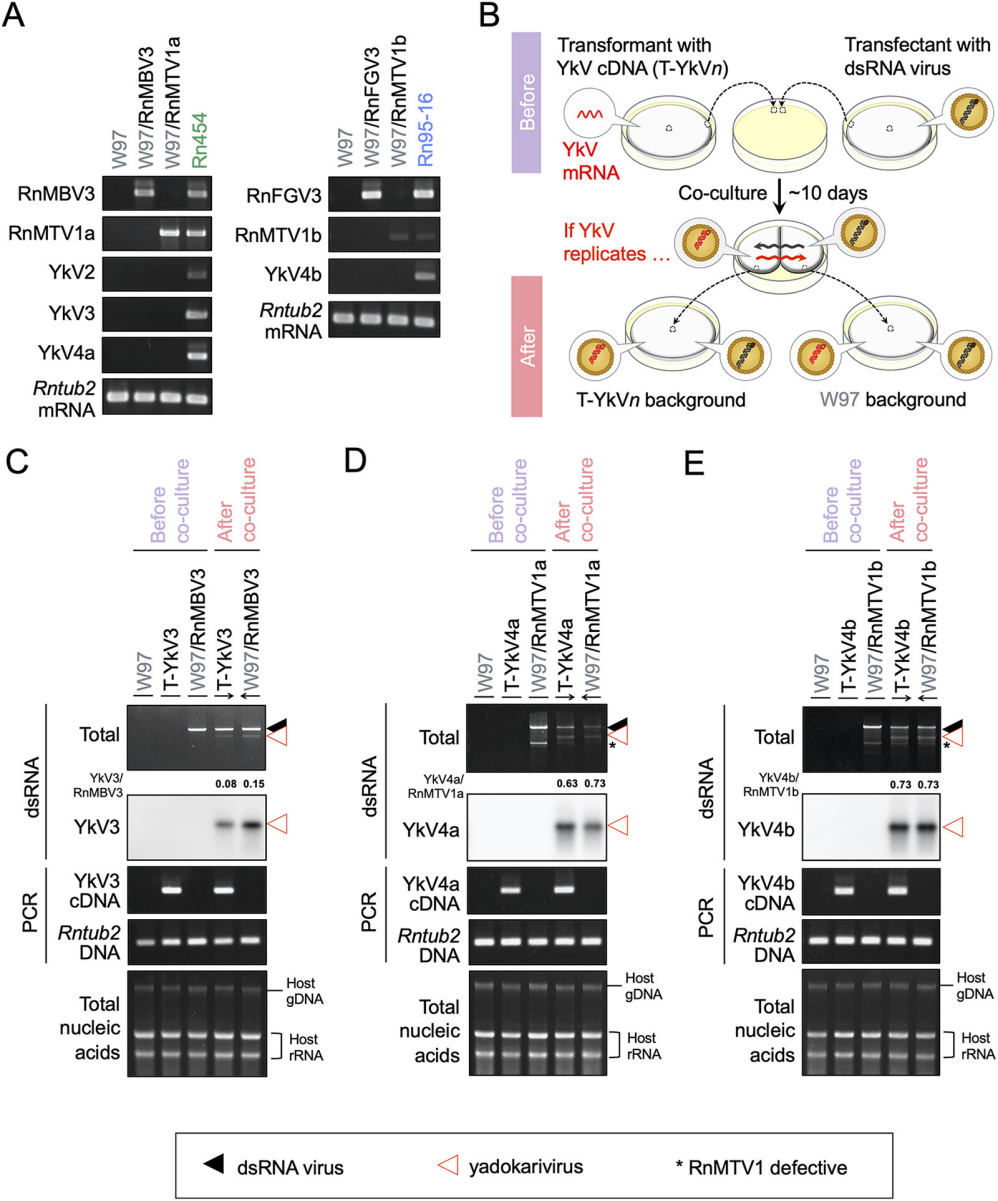
Identification of the partner of YkV3 and YkV4. (A) Confirmation of single virus infection in *R. necatrix* W97 transfected with RnMBV3, RnMTV1s, or RnFGV3 by RT-PCR. Strain W97 is a virus-free control. The field-collected fungal strains Rn454 and Rn95-16 as a source of the viruses were analyzed in parallel. *Rntub2* (*R. necatrix β*-tubulin gene) mRNA served as an internal control. (B) Experimental flow to identify dsRNA virus partners that support the replication of yadokariviruses. T-YkV*n* represents *R. necatrix* W97 transformed with an infectious cDNA clone of each yadokarivirus (where *n* refers to the numbers attached to the names of yadokariviruses). The transformants (T-YkV*n*) were cocultured for approximately 10 days with each of the transfectants harboring a single candidate dsRNA virus. Antibiotic resistant T-YkV*n* sides and antibiotic susceptible viral transfectant sides were each subcultured to examine for yadokarivirus replication and horizontal virus transfer. When yadokarivirus replication was supported, coculture allowed for mutually lateral virus transfer in both directions via hyphal fusions. If not, only dsRNA viruses could be transferred from the transfectant side to the transformant side. Yadokarivirus replicative form dsRNA was detected in *R. necatrix* subcolonies before and after the coculture between T-YkV*n* and the dsRNA virus transfectants. (C to E) The three coculture pairs were tested: the YkV3 cDNA transformant (T-YkV3) and the RnMBV3 transfectant (C); the YkV4a cDNA transformant (T-YkV4a) and the RnMTV1a transfectant (D); the YkV4b cDNA transformant (T-YkV4b) and the RnMTV1b transfectant (E). The top two panels show total or yadokarivirus dsRNA detected by ethidium bromide or Northern hybridization, respectively. The black triangles indicate dsRNA of RnMBV3 or RnMTV1s. The red triangles indicate dsRNA of YkV3 or YkV4s. The asterisks indicate defective dsRNA of RnMTV1. The values below the total dsRNA panel indicate the abundance ratio of the yadokarivirus dsRNA to the partner viral dsRNA measured by ImageJ in which the partner dsRNA intensity was expressed as 1. The middle two panels show PCR-based detection of the transgene of yadokariviruses cDNA and an internal control, respectively. A host β-tubulin gene (*Rntub2* DNA) was detected as an internal control. The bottom panel shows the electrophoretic profile of a portion of total nucleic acid (1 μg), the batch of which was used for dsRNA extraction (top two panels) and host genotyping (middle panels).

To identify the yadokarivirus-partner dsRNA viruses, we first attempted to isolate respective coinfecting viruses by a virion transfection method available for many fungal dsRNA viruses (26–28). Virion preparations were obtained from fungal strains Rn454, Rn95-16, and Rn430 and transfected into the Japanese *R. necatrix* standard strain W97 (29). After the screening of a series of transfectants, single infectants with five dsRNA viruses (RnMBV3, RnMTV1a, RnMTV1b, RnMTV1c, or RnFGV3) were established (Table 2). These five independent single infections by the viruses were confirmed by RT-PCR and dsRNA profiling (Fig. 2A and Fig. S2). These five dsRNA viruses were stably maintained in the W97 background, indicating that these viruses could independently complete their replication cycles without yadokariviruses, as in the case for YkV1’s partner YnV1. Importantly, no single infections with any yadokariviruses were obtained during virion transfection, suggesting their dependency on partner dsRNA viruses (data not shown).

**TABLE 2:** Transfectants and transformants of *R. necatrix* strain W97 used in this study

Strain name	Transfected virus or transformed construct	Reference
Transfectant		
W97/YnV1-A	YnV1-A from *R. necatrix* strain W1032	9
W97/RnMBV1	RnMBV1 from *R. necatrix* strain W779	This study
W97/RnMBV3	RnMBV3 from *R. necatrix* strain Rn454	This study
W97/RnMTV1a	RnMTV1a from *R. necatrix* strain Rn454	This study
W97/RnFGV3	RnFGV3 from *R. necatrix* strain Rn95-16	This study
W97/RnMTV1b	RnMTV1b from *R. necatrix* strain Rn95-16	This study
W97/RnMTV1c	RnMTV1c from *R. necatrix* strain Rn430	This study
Transformant		
T-YkV1	An infectious cDNA clone of YkV1	9
T-YkV3	An infectious cDNA clone of YkV3 whose 5′-terminal residue is U	This study
T-YkV4a	An infectious cDNA clone of YkV4a whose 5′-terminal residue is U	This study
T-YkV4b	An infectious cDNA clone of YkV4b whose 5′-terminal residue is C	This study

10.1128/mbio.01685-22.2 FIG S2 Recharacterization of viruses harbored in *R. necatrix* strains Rn454, Rn95-16, and Rn430. (A) Detection of viral RNA by RT-PCR. Host endogenous gene expression (*Rntub2* mRNA) was detected as an internal control. The *R. necatrix* strains Rn480 and Rn30 were included as positive controls for viral detection. (B) Detection of viral RNA by Northern hybridization of ssRNA-enriched total RNA. Host ribosomal RNA (rRNA) was detected as an internal control. (C) Comparative data showing the presence or absence of viruses in fungal strains Rn454, Rn95-16, and Rn430. The detection patterns for 2018 were reported by Arjona-Lopez JM, Telengech P, Jamal A, Hisano S, et al. (Environ Microbiol 20:1464–1483, 2018), while those in the current study are based on panels A and B and Fig. 2A. Among the viruses previously found from the two Spanish fungal strains Arjona-Lopez JM, Telengech P, Jamal A, Hisano S, et al. (Environ Microbiol 20:1464–1483, 2018), capsidless (+)ssRNA hypoviruses in the Rn454 and a fusagravirus (*Rosellinia necatrix* fusagravirus 2, RnFGV2) and capsidless (+)RNA fusagriviruses in Rn95-16 were undetectable in this study. Although Rn430 was supposed to be additionally infected by a fusagravirus strain (RnFGV3), this virus was also not detected in this study. These viruses possibly disappeared during subculture as reported elsewhere (Arjona-Lopez JM, Telengech P, Suzuki N, Lopez-Herrera CJ. Eur J Plant Pathol 158:111–119, 2020). (D) Electrophoretic profiles of total dsRNA purified from *R. necatrix* W97 singly transfected with RnMBV3, RnMTV1s, or RnFGV3. The virus source fungal strains (Rn454, Rn95-16, and Rn430) were analyzed in parallel. The bracket on the right side indicates the migration positions of the genomic dsRNA of RnMBV3, RnMTV1, and RnFGV3. The open red triangle indicates the migration positions of yadokarivirus dsRNA. The asterisk denotes the migration positions of defective dsRNA of RnMTV1. (E) Confirmation of single RnMTV1c infection of the *R. necatrix* W97 transfectant by RT-PCR. Strain W97 is a virus-free control. The field-collected fungal strain Rn430 is the RnMTV1c source in transfection. *Rntub2* (*R. necatrix β*-tubulin gene) mRNA served as an internal control. Download FIG S2, PDF file, 2.0 MB. Copyright © 2022 Sato et al. 2022 Sato et al. https://creativecommons.org/licenses/by/4.0/ This content is distributed under the terms of the Creative Commons Attribution 4.0 International license.

To examine the autonomous-replication ability or determine partner viruses of the yadokariviruses, full-length infectious cDNA clones to betayadokariviruses, YkV2, YkV3, YkV4a, and YkV4b were constructed, as in the case for YkV1 (9). These betayadokariviruses have sequence heterogeneity at the extreme 5′-terminal end of the plus-strand genomic RNA, either 5′-C/U or 5′-C/A (15) (Fig. 1B). Full-length cDNAs with either of the two residues at the terminus (see Table 2) were inserted into the dual-ribozyme cassette and were expected to produce authentic viral (+)RNA transcripts in transformed fungal cells. An exception was the infectious cDNA clone of YkV4b without any ribozymes. Each of the transformants, described as T-YkV*n* (here *n* refers to the serial numbers in each yadokarivirus) in the figures, were cocultured with each of the transfectants singly infected with a partner candidate dsRNA virus (Fig. 2B). After the coculture, mycelial plugs at the transformant side (T-YkV*n* side, antibiotic resistant) received a partner candidate and the transfectant sides (antibiotic susceptible) were each subcultured with or without antibiotics. Under the conditions where yadokarivirus can replicate, its replicative form dsRNA should be detected in both of the subcolonies from transformant and transfectant sides.

Among combinations of viruses from the fungal strain Rn454, only the coculture of transformants with YkV3 cDNA and transfectants with RnMBV3 resulted in YkV3 replication, showing mutual lateral transfer between the paired transformant and transfectant (Fig. 2C). Similarly, the dsRNA virus partners intrinsically hosting YkV4a and YkV4b in the fungal strain Rn454 or Rn95-16 were identified as RnMTV1a and RnMTV1b, respectively (Fig. 2D and E). These also clearly indicated the establishment of infectious cDNA clones to the betayadokariviruses YkV3, YkV4a, and YkV4b, which are able to replicate only in the presence of the respective partner dsRNA viruses. Although the full-length cDNA clone of YkV2 was prepared as the others, we failed to determine its partner dsRNA viruses, suggesting its noninfectivity for some reason or missing its partner dsRNA virus in Rn454 during the course of the research (data not shown).

The band intensity ratio of YkV1 replicative form dsRNA to YnV1 dsRNA has been observed to be approximately 1:1 or higher in the *R. necatrix* W97 background (9, 14). In contrast, the band intensity ratio of YkV3 dsRNA to RnMBV3 dsRNA was much smaller, approximately a tenth (Fig. 2C). That of YkV4a/b dsRNA to RnMTV1 dsRNA was around two-thirds to three-fourths (Fig. 2D and E). These results suggest that the accumulation ratio of yadokarivirus dsRNA to its partner virus dsRNA could vary depending on each pair.

### Partner swapping assay.

**FIG 3:**
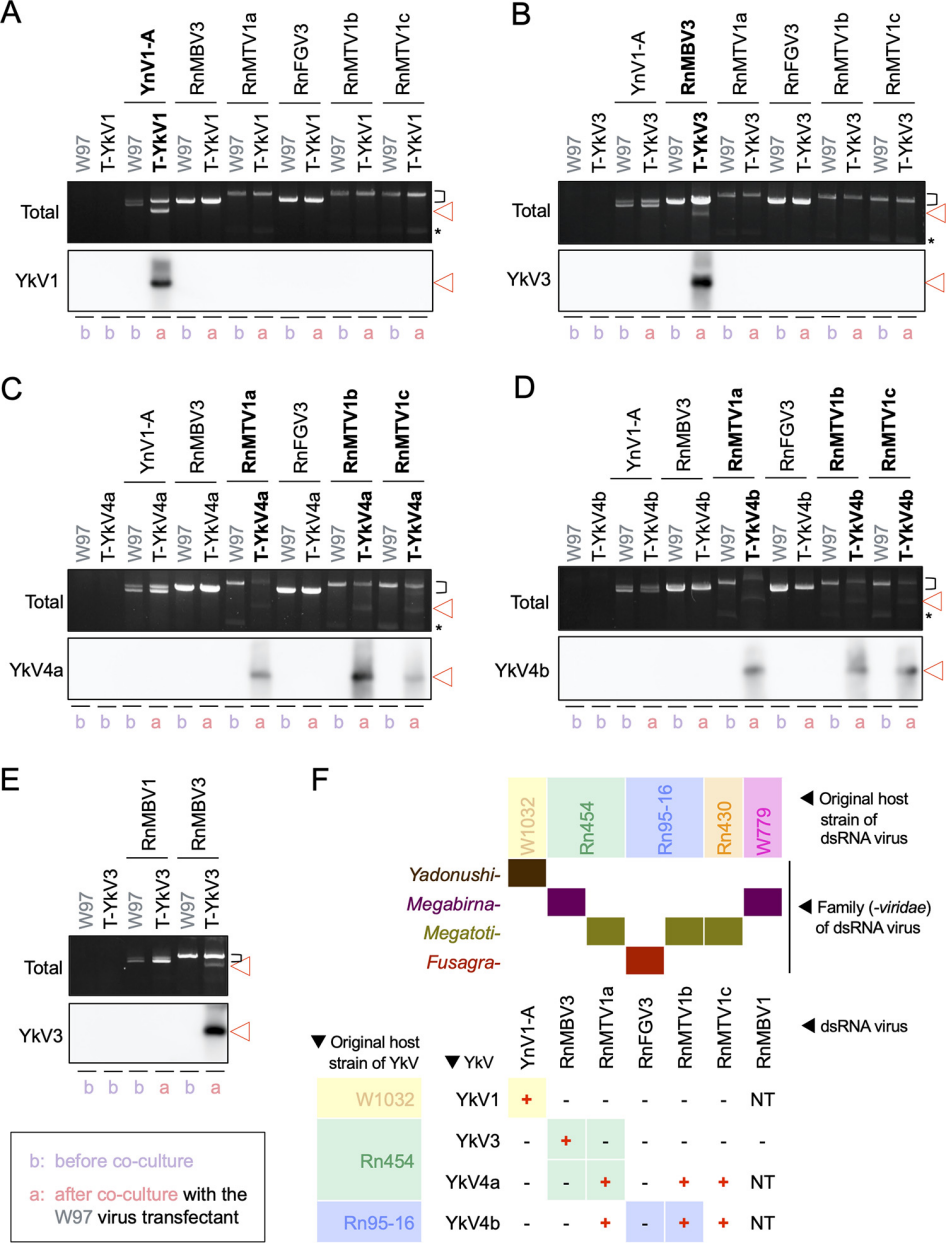
Swapping test of yadokariviruses and their partner dsRNA viruses. (A to E) Electrophoretic profile (top panel) and Northern blotting (bottom panel) of mycelial dsRNA to confirm yadokarivirus replication. Total dsRNA was purified from *R. necatrix* transformants with yadokarivirus cDNAs before (b) and after (a) the transfer of the partner dsRNA virus candidates. The top and bottom panels show total or yadokariviruses specific dsRNA, respectively. The transformant with each of the infectious cDNA clones of YkV1 (T-YkV1) (A), YkV3 (T-YkV3) (B and E), YkV4a (T-YkV4a) (C), and YkV4b (T-YkV4b) (D) received every possible partner candidate virus (YnV1-A, RnMBV3, three RnMTV1 strains, RnFGV3, or RnMBV1) via hyphal fusion (see Fig. 2B). Established partnership or yadokarivirus replication can be seen as their replicative form dsRNA of yadokariviruses. Original viral transfectants with the wild-type W97 genetic background, as well as virus-free W97 and its transformants before coculture, were used as negative controls for yadokarivirus detection. The brackets on the right side indicate the migration positions of dsRNA of the partner candidate dsRNA viruses. The red triangles indicate yadokarivirus dsRNA. The asterisks indicate defective dsRNA of RnMTV1. (F) Summary of the partner combinations. The plus and minus signs indicate the combinations with yadokarivirus replication supported or unsupported, respectively, in the *R. necatrix* strain W97 background. Colored cells behind the plus/minus signs indicate yadokariviruses and dsRNA viruses that were naturally coinfected in the single fungal strains. NT, not tested.

Along with the previously reported partnership (YkV1/YnV1), three additional partnerships (YkV3/RnMBV3, YkV4a/RnMTV1a, and YkV4b/RnMTV1b) were established in this study. To determine the partnership specificity, a swapping assay was carried out using fungal strains carrying single yadokarivirus infectious cDNA transgenes or dsRNA viruses as described above (Fig. 2B). The four established yadokarivirus cDNA transformants (T-YkV1, T-YkV3, T-YkV4a, and T-YkV4b) were cocultured with single transfectants with candidate partner dsRNA viruses, i.e., a megatotivirus, RnMTV1c isolated from Rn430, and a fusagravirus, RnFGV3 from Rn95-16, in addition to the above-mentioned YnV1, RnMBV3, RnMTV1a, and RnMTV1b. To examine the ability of each dsRNA virus to support yadokarivirus replication, production of the yadokarivirus replicative form dsRNA was monitored at the yadokarivirus cDNA transformant (T-YkV*n*) side, which received the dsRNA virus after the coculture. As a result, YkV1 replication was shown to be supported only by coinfecting YnV1 but not by the other dsRNA viruses tested (Fig. 3A). Similarly, YkV3 could be supported only by RnMBV3 but not by the other dsRNA viruses (Fig. 3B). YkV4a and YkV4b replication could not be assisted by YnV1, RnMBV3, or RnFGV3 but were supported by RnMTV1a, RnMTV1b, and RnMTV1c (Fig. 3C and D). Thus, partners for YkV4 and RnMTV1 were interchangeable among the strains within a species. In contrast, the partners for YkV1, YkV3, and YkV4 were not interchangeable among the three dsRNA viruses (YnV1, RnMBV3, and RnMTV1) that belong to the three independent families within the order *Ghabrivirales*.

The next question is whether yadokarivirus partners were interchangeable among the different viral species within the same family. To address this question, we used the megabirnavirus RnMBV1 (30) that was isolated from a Japanese strain (W779) of *R. necatrix* (Table 1) to compare the ability to support YkV3 replication with RnMBV3. Unlike monopartite RnMBV3, RnMBV1 has a bipartite genome (Fig. 1B). Although these viruses belong to the same family *Megabirnaviridae*, RdRP and CP of RnMBV1, respectively, show only 33.2% or 20.5% amino acid sequence identity to the counterparts of RnMBV3 (Fig. 1C). Here, we prepared *R. necatrix* strain W97 transfected with either of RnMBV1 wild-type (WT) or mutants that lacked the second segment (L2 in Fig. 1B) and harbored rearranged segments as previously reported (31) (Fig. S3A). When these viruses were transferred to the YkV3 cDNA transformant (T-YkV3), neither WT nor rearranged RnMBV1 supported YkV3 replication (Fig. 3E and Fig. S3B). These results suggest that the YkV3 partner was not interchangeable among the two different viral species in the same family.

10.1128/mbio.01685-22.3 FIG S3 Characterization of RnMBV1 transfectants and assay for their capacity to support YkV3 replication. (A) Electrophoretic profile of dsRNA from *R. necatrix* W97 transfected with RnMBV1. One wild-type (WT) strain (#B1) and three mutant transfectants (#A12, #A20, and #B22) were selected and analyzed, respectively. The original strains W779 and W97 were used as positive or negative controls for RnMBV1 detection, respectively. The top panel shows agarose gel electrophoresis patterns of total dsRNA isolated from the transfectants. The bottom two Northern panels show specific dsRNA bands hybridizing either of two genomic segments (L1 and L2) of RnMBV1. LS1 indicates rearranged L1 segment. Frequent rearrangement events of RnMBV1 dsRNA2 after transfection were reported earlier (Kanematsu S, Shimizu T, Salaipeth L, Yaegashi H, et al. Virology 450–451:308–315, 2014). The transfectant strain #B1 infected by wild-type RnMBV1 was used for the experiment in Fig. 3E. (B) Inability of RnMBV1 mutants to support YkV3 replication. The transformant of YkV3 cDNA (T-YkV3) was cocultured with each of the three transfectants with RnMBV1 mutants described above. YkV3 replication at the transformant side which received RnMBV1 mutants was tested. The original virus-free strain W97 was used as a negative control for YkV3 detection. T-YkV3 infected with RnMBV3 was used as a positive control for YkV3 replication. The top panel shows agarose gel of total dsRNA isolated from fungal colonies before and after coculturing with the W97 trasnfectant. The bottom three panels show Northern blots of total dsRNA with probes specific to YkV3, RnMBV3, and RnMBV1-L1, respectively. The open red triangles indicate the migration position of YkV3 dsRNA. Download FIG S3, PDF file, 0.7 MB. Copyright © 2022 Sato et al. 2022 Sato et al. https://creativecommons.org/licenses/by/4.0/ This content is distributed under the terms of the Creative Commons Attribution 4.0 International license.

Taken together, each of the tested yadokariviruses showed relatively narrow, within-species partnership specificity (Fig. 3F).

### Phylogenetic consideration on the partnerships.

**FIG 4:**
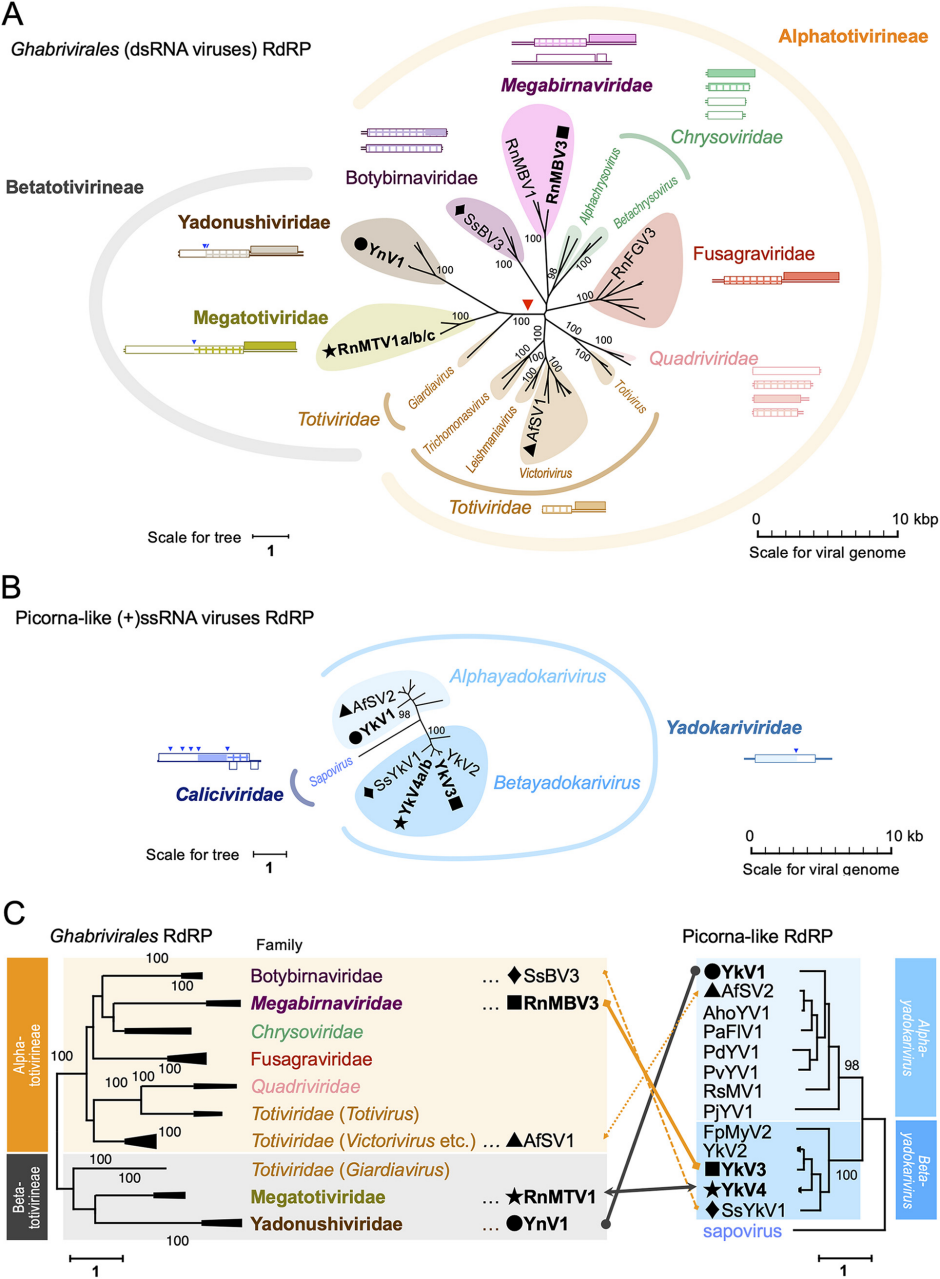
Phylogeny of the partnerships between yadokariviruses and their partners. (A and B) Maximum likelihood phylogenetic trees of RdRP of dsRNA viruses in the order *Ghabrivirales* (A) or RdRP of yadokariviruses with a sapovirus as an outgroup (B). The names of the established taxa are italicized, while those of the proposed taxa are not. Only names of the viruses discussed in this study, as well as associated genera and families are shown although other viruses were included in the analysis. The values flanking the nodes indicate bootstrap probabilities in 1,000 iterations. Bootstrap values lower than 95% and for nodes lower than genera (even though higher than 95%) were hidden. The original tree for the order *Ghabrivirales* with all the virus names and the bootstrap values is shown in Fig. S1. The partner pairs determined in this study or implied in previous reports (18, 22) are shown with the same symbols (●, ■, ★, ▴, or ♦). The red triangle indicates the branch between the proposed suborders “*Alphatotivirineae*” and “*Betatotivirineae*.” The genome organization of the exemplar virus for each family (NC_017990.1-NC_017991.1 for “*Botybirnaviridae*” (genus *Botybirnavirus*), NC_013462.1-NC_013463.1 for *Megabirnaviridae*, NC_007539.1-NC_007542.1 for *Chrysoviridae*, NC_013469.1 for “*Fusagraviridae*”, NC_016757.1-NC_016760.1 for *Quadriviridae*; NC_003745.1 for *Totiviridae*, LC333746.2 for “*Megatotiviridae*,” NC_040357.1 for “*Yadonushiviridae*”; NC_040360.1 for *Yadokariviridae*, and NC_006269.1 for *Caliciviridae*) is illustrated near the family name. The filled and lattice patterns represent RdRP and CP, respectively. The blue triangles indicate known polyprotein cleavage sites. (C) Partnership between yadokariviruses and dsRNA viruses depicted in the respective trees. The left side phylogenetic tree of members of the order *Ghabrivirales* was simplified from the original tree in panel A or Fig. S1. The right-side phylogenetic tree of yadokarivirus RdRPs is the same as that in panel B. Partner pairs are shown with the same symbols (●, ■, ★, ▴, or ♦) and connected by solid or dotted lines, respectively, for experimentally determined here or previously implied pairs. Abbreviations of yadokariviruses are explained in Table S1.

The partner dsRNA viruses determined in this study belong to the three phylogenetically distant families (“*Yadonushiviridae*,” *Megabirnaviridae*, and “*Megatotiviridae*”) in the order *Ghabrivirales* (Fig. 4A and Fig. S1). The order *Ghabrivirales* accommodates dsRNA viruses that have been reported from diverse hosts including animals, plants, fungi, oomycetes, and protozoa. The order *Ghabrivirales* can be divided into at least two phylogenetic mega-clades (e.g., see the “Toti-Chryso” virus clade in Shi et al. [2]) or the YnV1-involved phylogenetic tree in reference (10). Here, we propose the terms “*Alphatotivirineae*” and “*Betatotivirineae*” as the suborders in the *Ghabrivirales* (Fig. 4A and Fig. S1). Phylogenetic relationships among yadokariviruses (Fig. 4B) and those among their partner dsRNA viruses (Fig. 4A) did not correlate with their partnership specificities (Fig. 4C). An alphayadokarivirus YkV1 made a partnership with a yadonushivirus YnV1 that belongs to the suborder “*Betatotivirineae*”. The betayadokariviruses YkV4a and YkV4b also partnered with viruses in the suborder “*Betatotivirineae*,” a megatotivirus with three strains (RnMTV1a/b/c). In contrast, the other betayadokarivirus YkV3 made a partnership with a megabirnavirus RnMBV3 that belongs to the distinct suborder “*Alphatotivirineae*.” Thus, the partnership between yadokariviruses and partner dsRNA viruses is incongruent with their phylogenetic relationship.

### Heteroencapsidation of YkV3 and YkV4 replicative form dsRNA.

**FIG 5:**
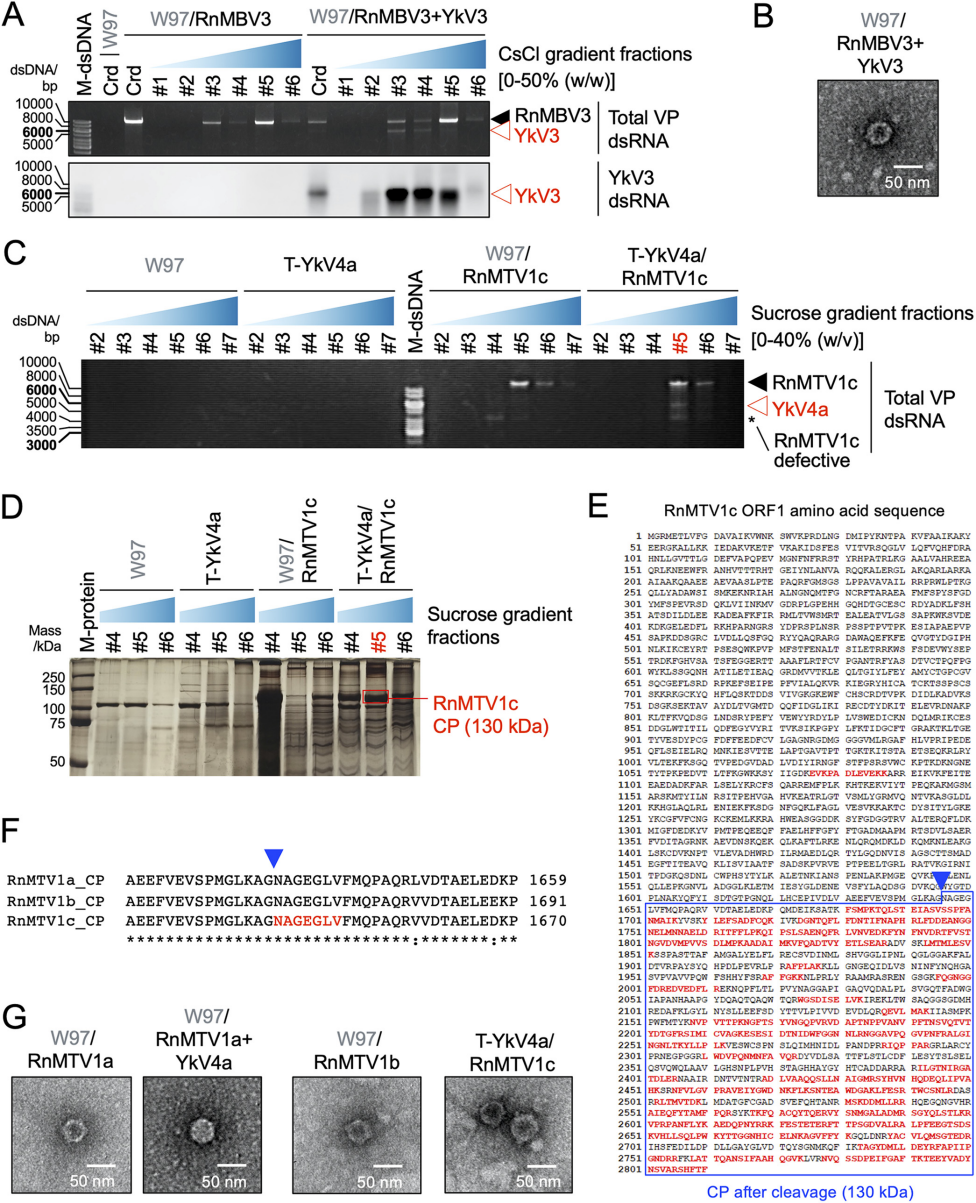
Heteroencapsidation of betayadokariviruses by partner dsRNA virus capsids. (A) Electrophoretic profile of dsRNA purified from virus particle (VP) fractions from *R. necatrix* infected by RnMBV3 alone or together with YkV3. VP dsRNA was extracted from crude virus particles (Crd) and those fractionated by cesium chloride (CsCl) buoyant density centrifugation. The top and bottom panels show total dsRNA stained by EtBr and YkV3 specific bands detected by Northern blotting, respectively. (B) Transmission electron microscopy (TEM) of the virus particles purified from the fungal strains coinfected by YkV3 and RnMBV3. (C) Electrophoretic profile of dsRNA purified from VP fractions from *R. necatrix* infected by RnMTV1c alone or together with and YkV4a. VP dsRNA was extracted from Crd and sucrose density gradient fractions. Virus-free W97 and T-YkV4a (W97 transformant with YkV4a cDNA), which did not show YkV4a replication in the absence of RnMTV1, were analyzed in parallel. (D) Silver staining of VP proteins electrophoresed in SDS-PAGE gel. The VP fractions of RnMTV1c and YkV4a above (A) were subjected to the analysis. M-protein refers to a protein size marker (Precision Plus Protein Dual Color Standards; Bio-Rad Laboratories, Inc.). The major protein band (around 130 kDa) enclosed in a red square was used for subsequent peptide mass fingerprinting (PMF) and N-terminal sequencing. (E) Result of the PMF analysis of the major 130-kDa protein in YkV4a/RnMTV1c virus particles. Peptide sequences identified by the PMF analysis were mapped to the amino acid sequence of RnMTV1c ORF1-encoding protein as denoted by the red letters. The blue triangle indicates the cleavage site determined by N-terminal sequencing of the 130 kDa protein band. (F) Alignment of the amino acid sequences of premature CP (the protein encoded by ORF1) of the three RnMTV1 strains. The blue triangle indicates the cleavage site determined in pnael E. (G) TEM images of the VP from *R. necatrix* infected by RnMTV1 strains with or without YkV4a.

We next confirmed whether YkV3 and YkV4 were encapsidated by the CPs of their partner dsRNA viruses. Virus particle (VP) fractions were obtained by cesium chloride or sucrose gradient ultracentrifugation and their dsRNA and protein components were analyzed (Fig. 5). Both YkV3 dsRNA (replicative form) of 5.7 kbp and RnMBV3 dsRNA of 9.0 kbp were cofractionated in higher buoyant density fractions (Fig. 5A). Electron microscopy of the fractions containing both dsRNAs showed spherical virus particles of around ~50 nm in diameter (Fig. 5B). This was expected from our previous observation that a sister virus of RnMBV3, RnMBV1, forms icosahedral particles of 52 nm with a *T *= 1 capsid (23). Similarly, both dsRNAs of YkV4a (5.3 kbp) and RnMTV1c (12.4 kbp) copurified in higher sedimentation velocity fractions (Fig. 5C). Sodium dodecyl sulfate (SDS)-PAGE of the VP fractions of YkV4a/RnMTV1c showed the major protein band corresponding to approximately 130 kDa (Fig. 5D). The major proteins of YkV4a/RnMTV1c were subjected to peptide mass fingerprinting (PMF) and N-terminal sequencing. The PMF analysis suggested that the peptide fragments were derived from the C-terminal part of the hypothetical protein encoded by RnMTV1c ORF1 (Fig. 5E). The N-terminal sequence of the 130 kDa RnMTV1c-CP was _1646_NAGEGLV… at map aa position 1646 to 1652 (Fig. 5E), which as conserved among the three RnMTV1 strains (RnMTV1a/b/c) (Fig. 5F). The three RnMTV1 strains with or without YkV4 formed spherical virus particles of around ~50 nm in diameter (Fig. 5G). This study first provides molecular characters of megatotiviriuses virions. In conclusion, these combined data suggest that the replicative form dsRNA of betayadokariviruses was also packaged in *trans* by the partner dsRNA viruses CP, as in the case of alphayadokarivirus YkV1.

### Effects of yadokariviruses on their partner viruses and host fungus.

**FIG 6:**
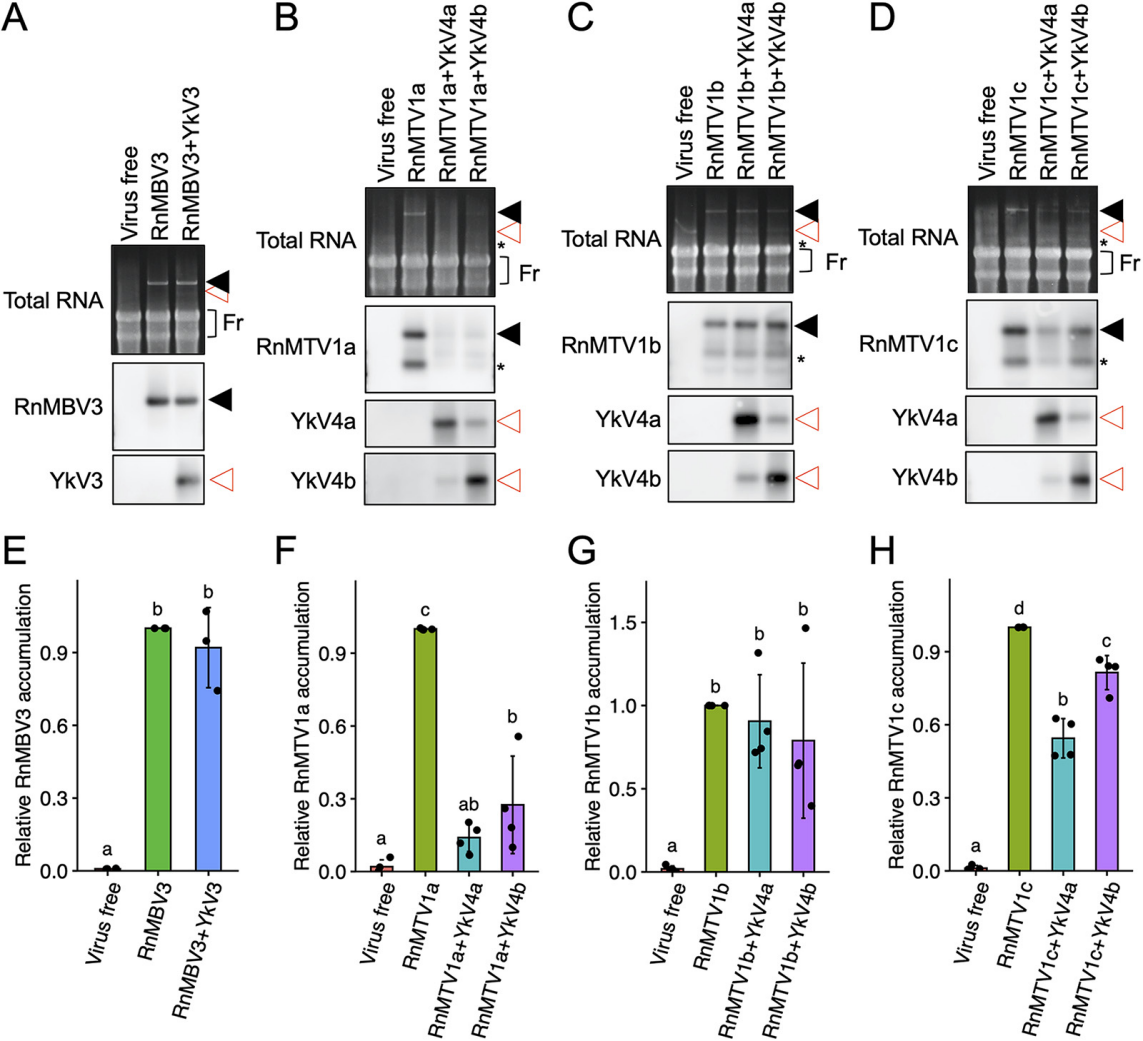
Effects of yadokariviruses on partner dsRNA viruses. (A to D) Viral RNA accumulation in *R. necatrix* W97 mycelia. The top panels show agarose gel patterns of total RNA (10 μg/lane) visualized with EtBr. “Fr” indicates fungal rRNA shown as a loading standard. The middle and bottom panels show Northern blots of total RNA for detecting partner dsRNA viruses and yadokariviruses. The filled black triangles indicate partner dsRNA virus RNAs. The open red triangles indicate yadokarivirus RNAs. Asterisks refer to defective RNA of RnMTV1. (E to H) Quantification of the partner dsRNA viruses. The band intensity of full-length RNA of each partner dsRNA virus (RnMBV3, RnMTV1a, RnMTV1b or RnMTV1c) under the presence or absence of the yadokariviruses was quantified from the repeated Northern blotting (Fig. 6A to D and Fig. S4A and S5A). The band intensity of each partner dsRNA virus in the singly infected sample on each Northern blot was defined as 1. The bar graphs show mean ± SD, while each plot shows the value obtained from each of the repeated experiments. The different letters above the bars represent statistical difference by Tukey’s test (*n* = 3 [E] or *n* = 4 [F to H], *P* < 0.05).

The previous study reported that YkV1 makes a mutualistic relationship with YnV1 by enhancing YnV1 accumulation (9). To investigate whether a similar relationship was observed in the currently revealed partnerships, the virus-free *R. necatrix* strain W97 was coinfected with the yadokariviruses and their partner dsRNA viruses via hyphal fusion as described in Fig. 2 to compare with the W97 solely infected with the dsRNA viruses. To this end, we compared the relative amount of viral RNA accumulated in fungal mycelia (Fig. 6 and Fig. S4A and S5A). YkV3 did not affect the accumulation of RnMBV3 (Fig. 6A and E and Fig. S4A). In contrast, YkV4a and YkV4b obviously decreased the accumulation of RnMTV1a (Fig. 6B and F and Fig. S5A). Interestingly, YkV4a and YkV4b showed, however, no or only milder negative effects on the accumulation of another RnMTV1 strain b (Fig. 6C and G and Fig. S5A). YkV4a and YkV4b exerted a mild negative effect, though at different degrees, on RnMTV1c (Fig. 6D and H and Fig. S5A). These results suggest that though YkV3 and YkV4 borrow capsids from the partner viruses, they give no benefits to the partner viruses at least in this experimental condition. Namely, YkV3 and YkV4 make commensal or parasitic relationships with their partner dsRNA viruses, unlike YkV1. Moreover, these relationships are variable even between similar viral strains, as suggested by the experiments using different YkV4 and RnMTV1 strains (Fig. 6B to D, and F to H and Fig. S5A) which share proteins with 84 to 99% amino acid sequence identity (Fig. 1C).

**FIG 7:**
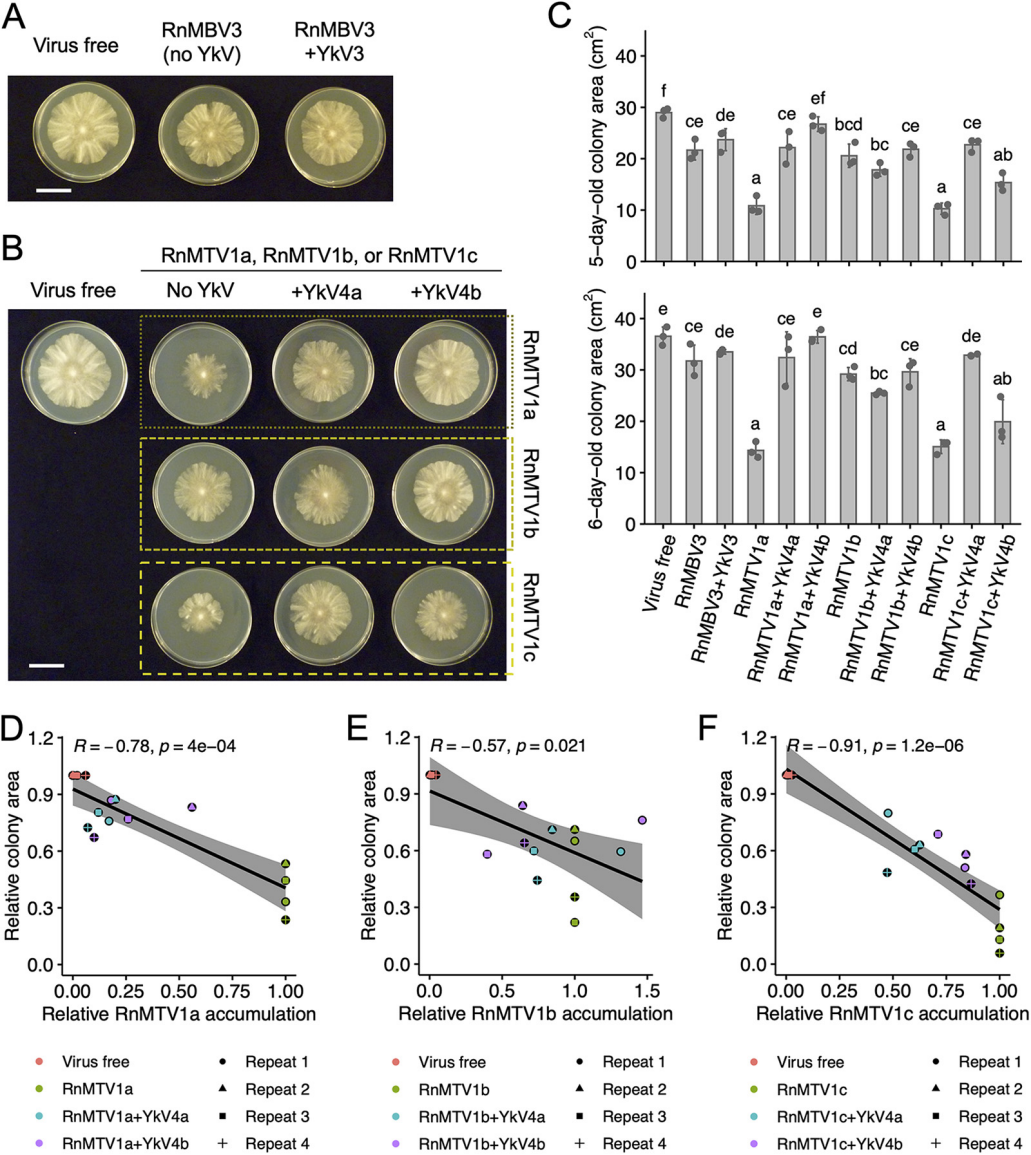
Effects of yadokariviruses on host fungus. (A and B) Colony morphology of virus-free and -infected *R. necatrix* W97. The RnMBV3-YkV3 pair is shown in panel A, while the RnMTV1-YkV4 pairs are shown in panel B. Fungal colonies were grown for 5 days on PDA media and photographed. The white bar represents 3 cm. (C) Colony size of virus-free and -infected *R. necatrix* W97. The top and bottom bar graphs show mean ± SD colony size of 5- or 6-day-old colonies, respectively. The plots indicate the size of colony subcultured individually on three plates in an experiment (Repeat 1). The different letters above the bars represent statistical differences by Tukey’s test (*n *= 3, *P* < 0.05). (D to F) Spearman’s correlation coefficient *R* between the relative accumulation of RnMTV1s and the relative fungal colony area. The data from four independent experiments were used. The raw images are shown in Fig. 6B to D and Fig. 7B for Repeat 1, and those in Fig. S5 are for Repeats 2 to 4. Relative RnMTV1 accumulation was estimated as in Fig. 6F to H. The relative fungal colony area was calculated by dividing the absolute values of virus-infected colonies by that of the virus-free colony on each picture. Different colors of spots indicate different RnMTV1 coinfection statuses, as shown at the bottom. The RnMTV1a-YkV4a/b pairs (D), the RnMTV1b-YkV4a/b pairs (E), or the RnMTV1c-YkV4a/b pairs (F) were separately analyzed. The shadow around the regression line indicates 95% confidence interval.

10.1128/mbio.01685-22.4 FIG S4 Repeated analyses to examine the effects of YkV3 on RnMBV3 and host fungus. (A) Viral RNA accumulation in the mycelia of *R. necatrix* W97. See the legend for Fig. 6A to D for abbreviations and symbols. The amount of total RNA loaded was 5 μg (Repeat 3) or 10 μg (Repeat 2 and the other experiments). (B) Colony phenotype of virus-free or -infected *R. necatrix* W97 on PDA media. The white bar represents 3 cm. For panels A and B, either 5- or 6-day-old cultures were photographed. (C) Spearman’s correlation coefficient (*R*) between the relative accumulation of RnMBV3 and the relative fungal colony area. The results of three independent experiments were analyzed. Relative RnMBV3 accumulation was estimated in Fig. 6E. The relative fungal colony area was measured from Fig. 7A and Fig. S4B, in which the absolute values for each virus-infected colony were normalized against that for virus-free. Different colors of spots indicate different RnMBV3 infection statuses, as shown on the right. Download FIG S4, PDF file, 0.2 MB. Copyright © 2022 Sato et al. 2022 Sato et al. https://creativecommons.org/licenses/by/4.0/ This content is distributed under the terms of the Creative Commons Attribution 4.0 International license.

10.1128/mbio.01685-22.5 FIG S5 Repeated analyses to examine the effects of YkV4s on RnMTV1s and host fungus. (A) Viral RNA accumulation in the mycelia of *R. necatrix* W97. See the legend for Fig. 6A to D for abbreviations and symbols. RnMTV1a/b/c and YkV4a/b were detected simultaneously by mixing DIG-labeled DNA probes for each viral strain at equal mass concentration. (B) Colony phenotype of virus-free or -infected *R. necatrix* W97 on PDA media. The white bar represents 3 cm. For each panel, either 5- or 6-day-old cultures were photographed. Download FIG S5, PDF file, 0.2 MB. Copyright © 2022 Sato et al. 2022 Sato et al. https://creativecommons.org/licenses/by/4.0/ This content is distributed under the terms of the Creative Commons Attribution 4.0 International license.

Next, we examined the effects of the viral infection on *in vitro* growth of the host fungus (*R. necatrix* strain W97) (Fig. 7A to C and Fig. S4B and S5B). Neither infection by RnMBV3 alone nor coinfection by RnMBV3 and YkV3 significantly affect the host fungal growth (Fig. 7A and C and Fig. S4B). In contrast, the three RnMTV1 strains (a, b, and c) retarded host fungal growth, although the extent of growth delay was different among the RnMTV1 strains (Fig. 7B and C and Fig. S5B). Notably, the amount of RnMTV1 accumulation was inversely correlated with the fungal colony size (Spearman’s rank correlation test, *P* < 0.05) (Fig. 7D to F), whereas the amount of RnMBV3 accumulation was not (Fig. S4C). YkV4 strains, which decreased RnMTV1 (a and c) accumulation, restored the fungal growth (Fig. 7B and C and Fig. S5B), almost reaching the level comparable to virus-free colonies at a maximum. Therefore, YkV4 parasitic to RnMTV1 was regarded as mutualistic to the host *R. necatrix*. Our study newly revealed that some yadokariviruses can be mutualistic to the host fungus, via interfering with the accumulation of their partner viruses pathogenic to the host fungus.

## DISCUSSION

The megataxonomy of viruses has recently been established and RNA viruses have been classified into five phyla within the kingdom *Orthornavirae*, the realm *Riboviria*. The five phyla include *Duplornaviricota* (dsRNA viruses), *Kitrinoviricota* [(+)ssRNA viruses], *Lenarviricota* [(+)ssRNA viruses], *Negarnaviricota* [(–)ssRNA viruses], and *Pisuviricota* [extended picorna-like superfamily, (+)ssRNA viruses and dsRNA viruses] (32, 33). The key players involved in the yado-kari/yado-nushi nature studied here belong to the phylum *Pisuviricota* (yadokariviruses, in the family *Yadokariviridae*, order *Yadokarivirales*) and *Duplornaviricota* (partner dsRNA viruses, order *Ghabrivirales*). The dsRNA viruses identified as hosting yadokariviruses in this and the previous study are YnV1 for YkV1 (9), RnMBV3 for YkV3, and RnMTV1 strains a to c for YkV4a/b (Fig. 1 and 3). Although YkV1, YkV3, and YkV4 were commonly isolated from *R. necatrix*, they show only modest levels of amino acid sequence identity (21% to 54%) in the hallmark of RNA viruses, RdRP (Fig. 1C). Although the dsRNA viruses hosting the yadokariviruses have a similar two-ORF genome architecture (Fig. 1B), they are more diverse from one another than yadokariviruses are to one another (Fig. 4) and show only low levels of RdRP sequence identity (interspecies level 14% to 22%) (Fig. 1C). BLASTP with the respective CP protein of the partner dsRNA viruses detected similar sequences only from members of the same virus family but not of the other families (data not shown). Particularly, proposed “*Yadonushiviridae*” and “*Megatotiviridae*” are distant from the family *Megabirnaviridae*, proposed “*Fusagraviridae*,” and other families. We propose to accommodate the former and latter groups each in the suborders “*Betatotivirineae*” and “*Alphatotivirineae*” (Fig. 4 and Fig. S1). The interstrain swapping results with YkV4 and RnMTV1 showing the interchangeability between their strains is not so surprising because the interstrain RdRP sequence divergence is 3.9% (between YkV4a and YkV4b) and 1.5% to 2.9% (among RnMTV1a, RnMTV1b, and RnMTV1c) (Fig. 1C). The partnership of each yadokarivirus established here is relatively virtuous; partners can be interchanged only between strains of a virus species, but not between virus strains of different species. However, the group of yadokariviruses collectively can promiscuously partner with diverse dsRNA viruses within the order *Ghabrivirales* that are phylogenetically distinct. This notion is strengthened by a few observations. Kozlakidis et al. (18) suggested that the alphayadokarivirus AfSV2 was encapsidated by Aspergillus foetidus slow virus 1 AfSV1, a member of the family *Totiviridae* (see Fig. 4 for their phylogenetic positions). Recently, Jia et al. (22) also reported a betayadokarivirus Sclerotinia sclerotiorum yadokarivirus 1 (SsYkV1) to be *trans*-encapsidated by Sclerotinia sclerotiorum botybirnavirus 3 (SsBV3), a biparticulate virus in the genus *Botybirnavirus* (see also Fig. 4). In addition, a plant capsidless (+)ssRNA virus (papaya meleira virus 2, umbravirus-like), belonging to the phylum *Kitrinoviricota*, is also thought to be *trans*-encapsidated by a coinfecting dsRNA virus, papaya meleira virus that is a member of the proposed family “*Fusgraviridae*” (34). This suggests that the yado-kari/yado-nushi nature exists in the kingdom Plantae. Another interesting notion is that betayadokarivurses YkV3 and YkV4 partner RnMBV3 and RnMTV1, belonging to the “*Alphatotivirineae*” and “*Betatotivirineae*,” respectively, although the alphayadokarivirus YkV1 is hosted by YnV1 belonging to the “*Betatotivirineae*” (Fig. 4). The other alphayadokarivirus AfSV2 and a betayadokarivirus SsYkV1 seem to be hosted by AfSV1 or SsBV3, respectively, belonging to the “*Alphatotivirineae*” (Fig. 4). This lack of correlation between the partnership specificities and the viral phylogeny implies that the partnerships between a yadokarivirus and a dsRNA virus might have been convergently acquired or lost.

Whether yadokarivirus RNA and RdRP are *trans*-encapsidated by the CP of their partner dsRNA virus is assumed to be the key first step for establishing their partnership (9) (Fig. 5). Since each yadokarivirus can establish a partnership only with a specific dsRNA virus (Fig. 3), there must be specific interactions between the CP or CP-RdRP of a dsRNA virus partner and the yadokarivirus RNA and/or RdRP. Also, the CP of a dsRNA virus homo-encapsidates its own RNA. Although no or few hints into possible yadokarivirus/partner specificity determinants are available, it is noteworthy that both the RnMBV3 5′ untranslated region (UTR) and the YkV3 3′ UTR have an identical 17-nucleotide sequence stretch, 5′-…AGCACAUAACGGCAAUU…-3′. However, no such sequence stretch is detectable in other yadokarivirus/dsRNA virus partner combinations. The best-studied dsRNA viruses in terms of packaging and assembly include Saccharomyces cerevisiae virus L-A (ScV-L-A), a totivirus within the family *Totiviridae* in the order *Ghabrivirales* (Fig. S1), which separately packages its own genomic dsRNA in addition to its satellite M1 dsRNA element encoding the yeast killer protein (35, 36). The satellite dsRNA has no sequence similarity to the ScV-L-A genomic dsRNA. The package signals identified on the genomic and satellite dsRNA have no sequence similarity but commonly contain a sequence stretch on the plus-strand RNA that could form a similar stem-loop structure with an A residue protruding on the 5′ stem (35). This package signal is recognized by the N terminal of the pol (RdRP) region of gag (CP)-pol molecules (CP-RdRP fusion protein), likely in a dimer form, translated by −1 ribosomal frameshifting (37). Yadokarivirus RNA packaging by the partner dsRNA virus CP may involve more potential players than ScV-L-A/M1 dsRNA packaging: yadokarivirus viral RNA (or viral replicative form) and RdRP, and partner CP and CP-RdRP fusion protein. It is reasonable to assume that yadokarivirus RdRP binds its own RNA, whether single stranded or double stranded, to catalyze transcription (synthesis of plus-strand RNA) or replication (synthesis of minus-strand RNA). As observed in ScV-L-A/M1, the partner CP-RdRP fusion protein of the partner dsRNA virus may bind a yadokarivirus RNA or RdRP molecule and facilitate CP-CP multimerization into capsid formation. This problem could be solved by establishing an *in vitro* or *vivo* package assay in which the assembly origin was uncoupled with *cis*-acting replication signals (37). A similar experimental design is necessary for yadokarivirus/dsRNA virus CP combinations.

A notable difference between newly and previously discovered partnerships is the ratio of contents of yadokarivirus dsRNA (replicative form) to that of its partner virus genomic dsRNA detected in purified particle preparations or mycelia (Fig. 2C to E and Fig. 5A and C). The YkV1 dsRNA accumulates more than or comparably to YnV1 dsRNA (9), while in this study all of the yadokariviruses tested showed much less accumulation than its corresponding partner virus dsRNA, based on the intensity ratio of dsRNA bands stained with ethidium bromide. What determines the dsRNA ratio of yadokarivirus to host dsRNA viruses remains elusive. A few steps may be critical for determining the ratio of the dsRNA accumulation of both viruses, such as homoencapsidation versus heteroencapsidation and replication/transcription efficiency in the homo- and heterocapsids, assuming that both of the two viruses replicate within particles.

**FIG 8:**
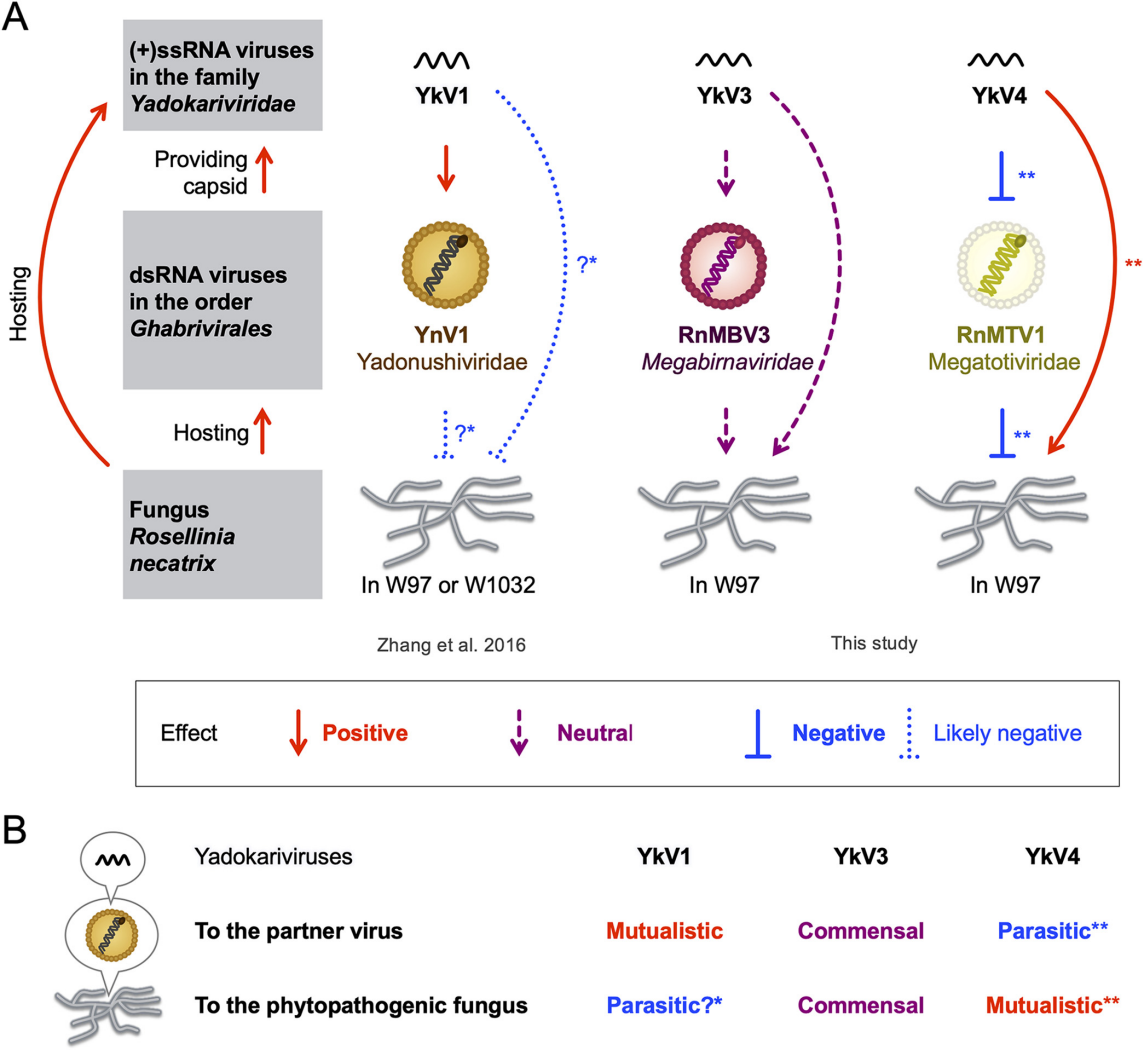
Illustration of the tripartite interactions. (A) The effects of yadokariviruses on their partner dsRNA viruses and the host fungus (*R. necatrix*). The effects of YkV1 on YnV1 and the host strain W97 or W1032 were suggested previously (9). The effects of YkV3 and YkV4 were analyzed in this study. *It remains to be investigated whether the negative effects of YkV1-YnV1 coinfection on the host growth (denoted by a quotation mark) were exerted by both or either of the viruses. **The degree of YkV4 effects varied, from negative (inhibitory) to neutral, depending on both YkV4 and RnMTV1 strains. (B) Summary of symbiotic relationships between yadokariviruses and their partner dsRNA viruses or the host fungus. Whether yadokariviruses are mutualistic/commensal/parasitic is different among the three different yadokarivirus/dsRNA virus partner/host fungus systems. * and **Same is in panel A.

This study showed that yadokariviruses make diverse patterns of mutualistic/commensal/parasitic relationships with partner dsRNA viruses and their host fungus (Fig. 6 and 8). The previous study (9) suggested that YkV1 enhances YnV1 accumulation in *R. necatrix* strain W97 and that coinfection of YkV1 and YnV1 in *R. necatrix* strain W1032 is associated with a fungal growth defect (9) (Fig. 8A). Namely, YkV1 was mutualistic to its partner virus and appeared to be parasitic to the host fungus (Fig. 8B). In contrast, YkV3 showed no apparent effects on the accumulation of RnMBV3 (Fig. 6A and E, and Fig. S4A) and the growth of *R. necatrix* W97 (Fig. 7A and C and Fig. S4B), although YkV received benefits (heterocapsid and replication materials) from both its partner virus and the host fungus (Fig. 8A). That is, YkV3 can be regarded as commensal to RnMBV3 and host fungus (Fig. 8B). YkV4a and YkV4b, on the other hand, decreased the accumulation of RnMTV1a (Fig. 6B and F and Fig. S5A), which rescued the host *R. necatrix* W97 from the RnMTV1a-mediated growth inhibition (Fig. 7B and C and Fig. S5B). That is, YkV4a and YkV4b were parasitic (harmful) to their partner virus RnMTV1a but mutualistic (beneficial) to the host fungus infected by RnMTV1a (Fig. 8). The degree of this YkV4’s parasitic effect on RnMTV1 differed between the combinations of YkV4 and RnMTV1 strains. YkV4a and YkV4b showed none or milder negative effects on the accumulation of RnMTV1b (Fig. 6C and G and Fig. S5A) or host growth (Fig. 7B and C and Fig. S5B). How much YkV4 decreased RnMTV1c accumulation was different between the YkV4 strains. Compared to YkV4b, YkV4a tended to have a greater negative impact on RnMTV1c (Fig. 6D and H and Fig. S5A). Thus, the relationships between yadokariviruses and partner viruses can change even between similar viral strains with high levels of amino acid sequence identity approximately 90% (Fig. 1C), evoking great interest in the evolutionary orientation of the yadokariviruses parasitism/mutualism. Although fungal viruses, which reduce the virulence of phytopathogenic fungi, have potential as biocontrol agents (38), this study shows that hypovirulence effects could be canceled by multilayered virus-virus interactions, as exemplified by the relationship between YkV4 and RnMTV1. The molecular mechanisms that govern the yadokariviruses mutualistic/parasitic nature will be of future challenge. The competition between yadokariviruses and their partner viruses for the same viral factor (capsids) might explain the parasitic effect of some yadokariviruses on their partner virus.

This study substantiated the widespread yado-kari nature of diverse yadokariviruses and their partnership specificity, building up a solid platform for further studying the virus neo-lifestyle (yado-kari/yado-nushi nature) or the three-layered virus (capsid borrower)-virus (capsid donor)-host interaction.

## MATERIALS AND METHODS

### Fungal strains and growth conditions.

The virus and fungal strains used in this study are listed in Table 1 (for natural isolates) and Table 2 (for transformants and transfectants), except for *R. necatrix* strains Rn480 and Rn30. The fungal strains Rn480 and Rn30 (15) were used as a positive control for the detection of hypovirus-like sequences, RnFGV2, a fusagravirus (RnFGV2), or fusarivirus-like sequences (Fig. S2). The fungal strains were subcultured on Difco potato dextrose agar (PDA) medium (Becton, Dickinson and Co.) in shading state at room temperature (around 25°C).

### Sequencing of the viral genome.

The full-length genome sequence of RnMBV3 and RnMTV1b, previously only partially determined by next-generation sequencing (NGS) (15), was completed in this study. The termini of the viral dsRNA were sequenced by RNA ligase mediated RACE (RLM-RACE), as previously described (15, 39). The nonsegmented nature of RnMBV3 genome was reconfirmed by Sanger sequencing of 103 cDNA fragments randomly prepared from dsRNA of *R. necatrix* W97 singly transfected with RnMBV3. The random cDNA library was prepared as previously described (15, 40). The full-length genome sequence of the newly found RnMTV1 strain named a (RnMTV1a) was determined by genome walking analysis (41) with the other RnMTV1 sequences as a reference, and RLM-RACE, using dsRNA fraction purified from *R. necatrix* W97 singly transfected with RnMTV1a. For RT-PCR of these viral fragments, M-MLV Reverse Transcriptase (Invitrogen, Thermo Fisher Scientific Inc.) and ExTaqHS (TaKaRa Bio Inc.) were used. The amplified fragments cloned into the pGEM-T Easy Vector (Promega Corp.) were sequenced after amplification by PCR with Quick *Taq* HS DyeMix (TOYOBO Co., Ltd.). Sanger sequencing was performed by a BigDye Terminator v3.1 Cycle Sequencing kit (Thermo Fisher Scientific Inc.) with 3130xl DNA Analyzers (Applied Biosystems, Thermo Fisher Scientific Inc.). The sequences of virus-specific primers used for the PCRs are available upon request.

### Phylogenetic analysis.

Phylogenetic relationships among yadokariviruses and dsRNA viruses were separately analyzed based on the deduced amino acid sequence of their RdRPs listed in Table S1 and Table S2. Amino acid sequence alignment was performed by MAFFT version 7.490 (https://doi.org/10.1093/molbev/mst010) with the method L-INS-i (42). Ambiguously aligned sites were trimmed by trimAl version 1.4 with the option “automated1” (43). The maximum likelihood phylogenetic trees were generated based on the trimmed sequence alignment by IQ-TREE version 2.0.3 with the best fit models (“LG+F+R5” for *Ghabrivirales* and “VT+I+G4” for *Yadokariviridae*) and the ultrafast bootstrap method in 1,000 iterations (44). The phylogenetic tree was visualized using MEGA X (45).

10.1128/mbio.01685-22.7 TABLE S2 Viruses in the order *Ghabrivirales* used for phylogenetic analysis. Download Table S2, DOCX file, 0.04 MB. Copyright © 2022 Sato et al. 2022 Sato et al. https://creativecommons.org/licenses/by/4.0/ This content is distributed under the terms of the Creative Commons Attribution 4.0 International license.

The global identity of the amino acid sequence of viral proteins was estimated by Clustal Omega version 1.2.4 with default settings (46). The full names of the viruses and accession numbers of the viral proteins used for the analysis are listed in Table S3. The heatmap was drawn using the R package “ggplot2” version 3.3.5 (https://cran.r-project.org).

### Purification and analyses of virus particles.

Crude virus particle fractions were purified from *R. necatrix* by ultracentrifugation as previously described (30) with slight modifications. Sucrose cushion (20%) in the ultracentrifugation to obtain crude extracts of virus particles was omitted for Fig. 5A and B. Crude virus particle fractions were further subjected to 10% to 50% (wt/wt) CsCl (for Fig. 5A and B) or 10% to 40% (wt/vol) sucrose (for Fig. 5C to G) density gradient centrifugation. The CsCl and sucrose gradient centrifugations were performed at ~210,000 × *g* for 2 h at 16°C or 4°C, respectively. After the gradient centrifugation, six CsCl or seven sucrose gradient fractions were obtained from top to bottom, numbered in ascending order according to increased densities. Viral dsRNA (including replicative form dsRNA) in the crude extracts and each of the fractions was extracted by a cellulose affinity column chromatography method as described below. The fractions containing viral dsRNA were diluted with 50 mM sodium phosphate buffer (pH 7.0) and further centrifuged to obtain concentrated pure virus particles. The purified virus particles were stained with EM stainer (Nissin EM Co.) and observed by a Hitachi H-7650 electron microscope. The purified virus fractions were subjected to SDS-PAGE followed by silver staining as previously described (47, 48). Peptide mass fingerprinting (PMF) and N-terminal sequencing of a major protein band on SDS-PAGE gel were also performed as previously described (47).

### Vector construction.

Full-length cDNAs of YkV3 and YkV4a were synthesized by outsourcing (GENEWIZ, Inc.) (see Table 2). These cDNAs were attached with the hammerhead ribozyme (HHRz) and the hepatitis delta virus ribozyme (HDVRz) at the 5′-terminal and 3′-terminal, respectively, and cloned into the *Not* I site (between glyceraldehyde-3-phosphate dehydrogenase gene promoter and terminator from *Cryphonectria parasitica*) of pCPXHY3 (49). The infectious cDNA clone of YkV4b was cloned manually and not attached with ribozymes exceptionally. The 5′- and 3′-half parts of YkV4b were each amplified by RT-PCR. The reverse transcription and PCR were performed by M-MLV Reverse Transcriptase (Invitrogen, Thermo Fisher Scientific Inc.) and ExTaqHS (TaKaRa Bio Inc.), respectively. The two amplicons were separately cloned into pGEM-T easy (Promega Corp.). By using these subcloned vectors as a template, the 5′- and 3′-half parts of YkV4b cDNA were secondarily amplified for subsequent in-fusion cloning reaction. The two amplified fragments were simultaneously cloned into the *Not* I site of pCPXHY3 by the In-Fusion HD Cloning kit (TaKaRa Bio Inc.). The sequences of primers used for the PCRs are listed in Table S4.

10.1128/mbio.01685-22.9 TABLE S4 Primers used for cloning of the infectious cDNA clone of YkV4b. Download Table S4, DOCX file, 0.03 MB. Copyright © 2022 Sato et al. 2022 Sato et al. https://creativecommons.org/licenses/by/4.0/ This content is distributed under the terms of the Creative Commons Attribution 4.0 International license.

### Transfection, transformation, and hyphal fusion of *R. necatrix*.

Spheroplasts of the *R. necatrix* virus-free strain W97 were prepared as previously described (50). Plasmid transformation and viral virion transfection were performed as previously described (50, 51) with modification of the regeneration media from potato dextrose broth (Becton, Dickinson and Co.) glucose to YPM (1% [wt/vol] yeast extract, 2% [wt/vol] Bacto peptone, and 0.6 M mannitol) and from YCDA to YPDMA (0.2% [wt/vol] yeast extract, 0.2% [wt/vol] Bacto peptone, 1% [wt/vol] glucose, 0.6 M mannitol, and 1.5% [wt/vol] agar) (14). To establish the transfectants singly infected by each virus, strain W97 was first transfected with virus particles purified from *R. necatrix* strains Rn454, Rn95-16, Rn430, or W779. In the cases where the single-virus transfectants were not obtained, strain W97 was secondarily transfected with virus particles purified from the first viral transfectants of W97. The mRNA expression from the transformed full-length yadokarivirus-cDNAs was confirmed by RT-PCR.

The transformants carrying full-length yadokarivirus-cDNAs were inoculated with the dsRNA viruses infected W97 via hyphal fusion (52). Mycelial plugs of each transformant and transfectant were placed on PDA 5 to 10 mm apart from each other and cocultured for around 10 days. The transformant side was selected by subculture on PDA containing appropriate antibiotics (0.2 μg/ml benomyl or 40 μg/ml hygromycin B for transformants carrying pCPXBn or pCPXHY3, respectively). The transfectant side was selected by subculture on normal PDA, followed by confirmation that it did not grow on PDA containing antibiotics. The viral lateral transfer was first detected by mycelial direct RT-PCR, which was originally reported by Urayama et al. (53) and modified by Sato et al. (54). Virus replication was further confirmed by viral dsRNA accumulation in the fungal hosts.

### Nucleic acid extraction.

Total nucleic acids were extracted from *R. necatrix* cultured on PDA-cellophane for 3 to 5 days. Frozen mycelial powder was suspended in STE-SDS buffers (55) or 50 mM NaCl, 100 mM Tris-HCl (pH 8.0), 10 mM EDTA (pH 8.0), and 0.5% SDS. Proteins were removed by phenol-chloroform-isoamylalcohol (PCIA) extraction followed by phenol clarification with chloroform-isoamylalcohol (CIA). The supernatant was used for the extraction of ssRNA, dsRNA, total RNA, or genomic DNA.

For ssRNA enrichment (56), the total nucleic acids in STE-SDS buffer were mixed with a one-fifth volume of 10 M lithium chloride and kept on ice for 2 to 3 h. The RNA pellets were rinsed with 70% ethanol, dried up, and dissolved in sterilized milli-Q water. The crude RNA was used for Northern hybridization. For RT-PCR, a constant amount of the extracted RNA was further treated with RQ1 RNase-Free DNase (Promega Corp.). After the treatment, DNase was removed with PCIA and CIA. The ssRNA was collected by 2-propanol or ethanol precipitation with sodium acetate and rinsed with 70% ethanol. The pellets were dried and suspended into milli-Q water to use as the templates for RT-PCR.

For total RNA and dsRNA extraction, the total nucleic acid in STE-SDS buffer was first precipitated using 2-propanol and sodium acetate. The pellets were rinsed with 70% ethanol, dried up, and dissolved in sterilized milli-Q water. To obtain total RNA, a constant amount of the total nucleic acids was treated with RQ1 RNase-Free DNase (Promega Corp.), followed by the clarification as described above. To obtain crude dsRNA, a constant amount (100 to 300 μg, optimized for each virus or experiment) of the total nucleic acids was subjected to cellulose (50 mg of cellulose powder B; Advantec Co., Ltd.) column chromatography as previously described (57). The extracted dsRNA was directly used for electrophoresis or further treated with RQ1 DNase (Promega Corp.) and S1 Nuclease (Invitrogen, Thermo Fisher Scientific Inc.). The DNase and S1 nuclease were removed as described above.

For genomic DNA extraction, the total nucleic acids suspension was treated with 10 μg/ml RNase A (Sigma-Aldrich Co., LLC) at 37°C for 30 min. The enzyme was removed by PCIA and CIA extractions. Genomic DNA in the supernatant was precipitated with 2-propanol and sodium acetate and then rinsed with 70% ethanol. Dried pellets were dissolved into TE buffer and used for genotyping (see below).

### RT-PCR and PCR-based genotyping.

Toward viral RNA detection by RT-PCR, the ssRNA-enriched fractions described above were used for cDNA synthesis by M-MLV Reverse Transcriptase (Invitrogen, Thermo Fisher Scientific Inc.) with the random primer [hexadeoxyribonucleotide mixture; pd (N)_6_] (TaKaRa Bio Inc.) according to the manufacturer’s instruction in a half scale. The cDNA was used as a template for PCR by Quick *Taq* HS DyeMix (TOYOBO Co., Ltd.) on a 10-μl scale. PCR-based genotyping of *R. necatrix* was also performed by Quick *Taq* HS DyeMix (TOYOBO Co., Ltd.) on a 10-μl scale with the host genomic DNA template. Primers are listed in Table S5.

10.1128/mbio.01685-22.10 TABLE S5 Primers used for RT-PCR, DIG-labeling PCR, or genotyping. Download Table S5, DOCX file, 0.04 MB. Copyright © 2022 Sato et al. 2022 Sato et al. https://creativecommons.org/licenses/by/4.0/ This content is distributed under the terms of the Creative Commons Attribution 4.0 International license.

The RT-PCR and genomic PCR products were electrophoresed in 1% (wt/vol) agarose in 0.5× TAE. The nucleic acids were visualized with ethidium bromide (EtBr) by postgel staining. GeneRuler 1-kb DNA ladder (Thermo Fischer Scientific, Inc.) (described as “M-dsDNA” in the figures) was constantly used as a molecular size marker for nucleic acids.

### Electrophoresis and Northern hybridization of RNA.

Northern hybridization of the ssRNA-enriched fractions (10 μg per lane) was performed via the MOPS-formaldehyde denaturing system as previously described (58). Northern hybridization of total RNA (10 μg per lane) and dsRNA (purified from a constant amount of total nucleic acids, see above) was performed via an alkaline-denaturing system as follows. After the agarose gel electrophoresis of the total RNA or dsRNA in 0.5× TAE or 0.5× TBE as described above, the gels were denatured in 0.1 N NaOH for 30 min. The alkalized gels were neutralized by immersing in 1.5 M Tris-HCl (pH 7.5) and 0.5 M NaCl for 15 min twice. Denatured dsRNA on the gel was blotted onto a nylon membrane (Hybond-N+; GE Healthcare, Inc.) by a standard capillary method with 20× SSC (3 M NaCl and 0.3 M sodium citrate) overnight. Transferred dsRNA was fixed to the membrane by an UV cross-linker at 240,000 μJ/cm^2^. Specific dsRNA bands were detected with cDNA probes labeled with digoxigenin-11-dUTP (DIG) and the antibody for DIG (Anti-Digoxigenin-AP, Fab fragments; F. Hoffmann-La Roche, Ltd.) according to the manufacturer’s instruction (F. Hoffmann-La Roche, Ltd.). The DIG-labeled cDNA probes were prepared by PCR DIG Labeling Mix (F. Hoffmann-La Roche, Ltd.) with the primers listed in Table S5. The template for the DIG-labeling PCR was viral cDNAs subcloned into plasmid vectors, i.e., yadokariviruses cDNA cloned into pCPX vectors as described above, or other viral cDNAs synthesized by PrimeScript One Step RT-PCR kit Ver.2 (TaKaRa Bio Inc.) and cloned into pGEM-T Easy (Promega Corp.).

### Quantification and statistical analyses.

The intensity of dsRNA bands was quantified by ImageJ version 1.52a according to the provider’s instructions (https://imagej.nih.gov/nih-image/manual/tech.html).

The fungal colony size was also quantified by ImageJ. Three independent subcultures were prepared for every virus-free or -infected fungal strain. The experiments were repeated three or four times. We first converted the original RGB color pictures to 16-bit grayscale and then selected each colony with the tracing tool to measure the area.

The statistical differences among the virus-free and -inoculated fungal strains were analyzed by Tukey’s test (*n *= 3 or 4; *P* < 0.05) using the R package “multcomp” version 1.4.8 (59). The bar graphs with scatterplots were generated using the R package “ggplot2” version 3.3.5 and “ggpubr” version 0.4.0.999 (https://cran.r-project.org). The mean and standard deviation (SD) were calculated by “ggpubr.”

The correlation of fungal colony sizes (normally distributed) with the accumulation of partner dsRNA viruses under the presence or absence of yadokariviruses (nonnormally distributed) was analyzed by Spearman’s rank correlation test by “ggpubr.”
