# Supplementary figures and images for: Three-Layered Complex Interactions among Capsidless (+)ssRNA Yadokariviruses, dsRNA Viruses, and a Fungus
Source: mBio. 2022 Aug 30;13(5):e01685-22. doi: 10.1128/mbio.01685-22 (PMC9600902; doi:10.1128/mbio.01685-22)

Figure S1

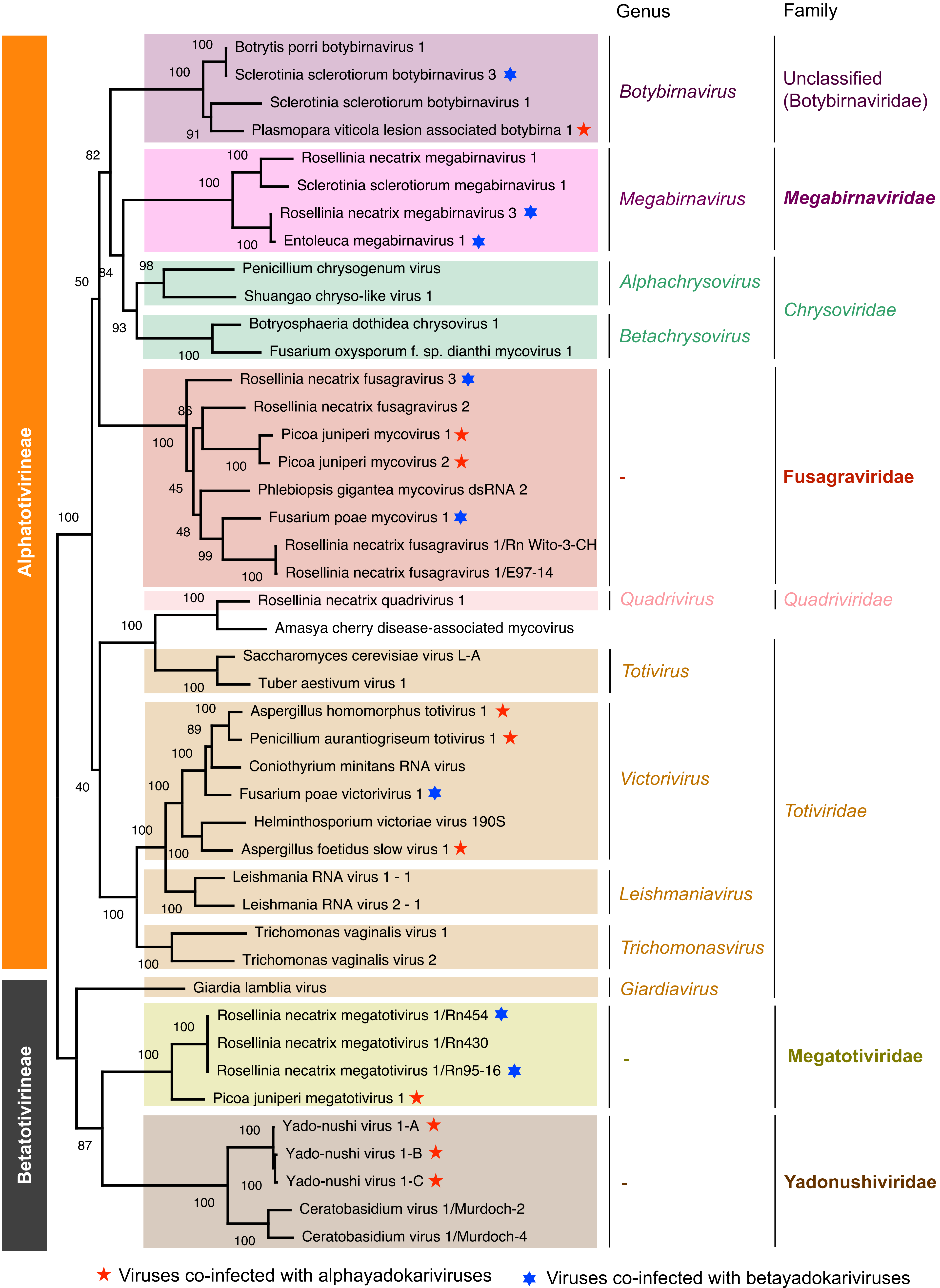

Supplement: FIG S1 [file mbio.01685-22-s0001.pdf]

Figure S2

A

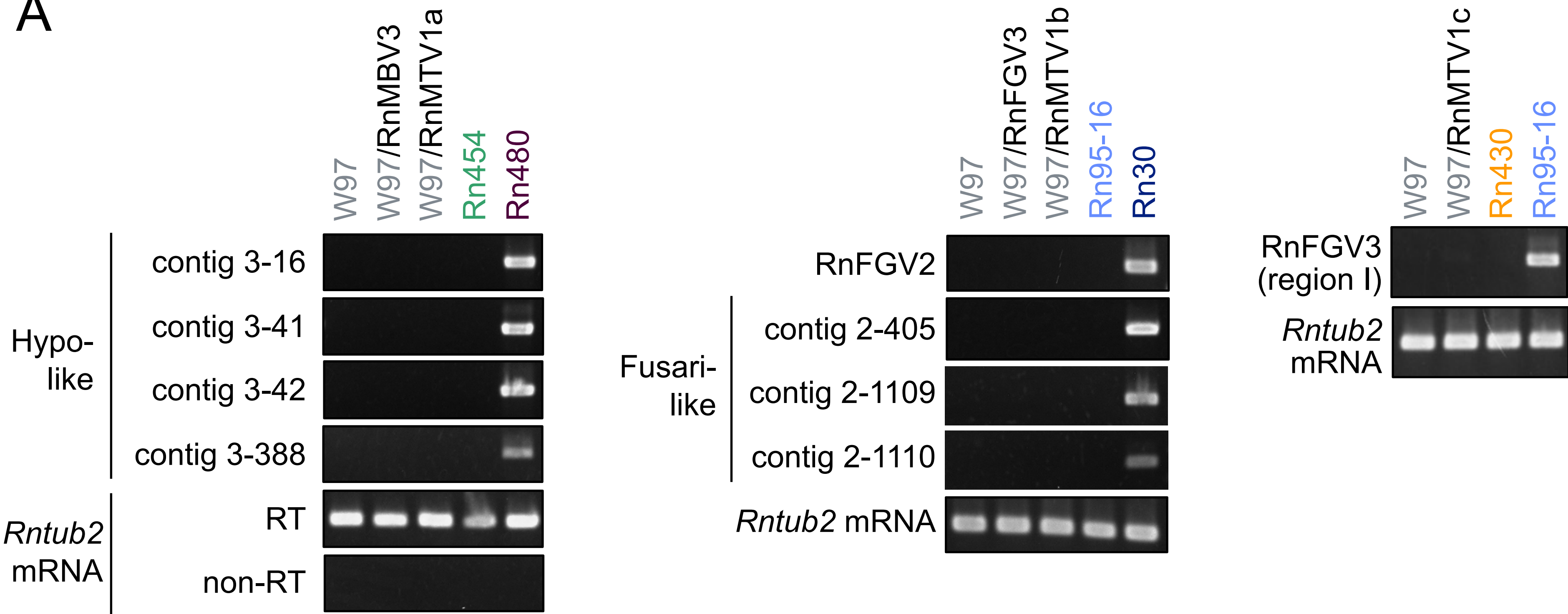

B

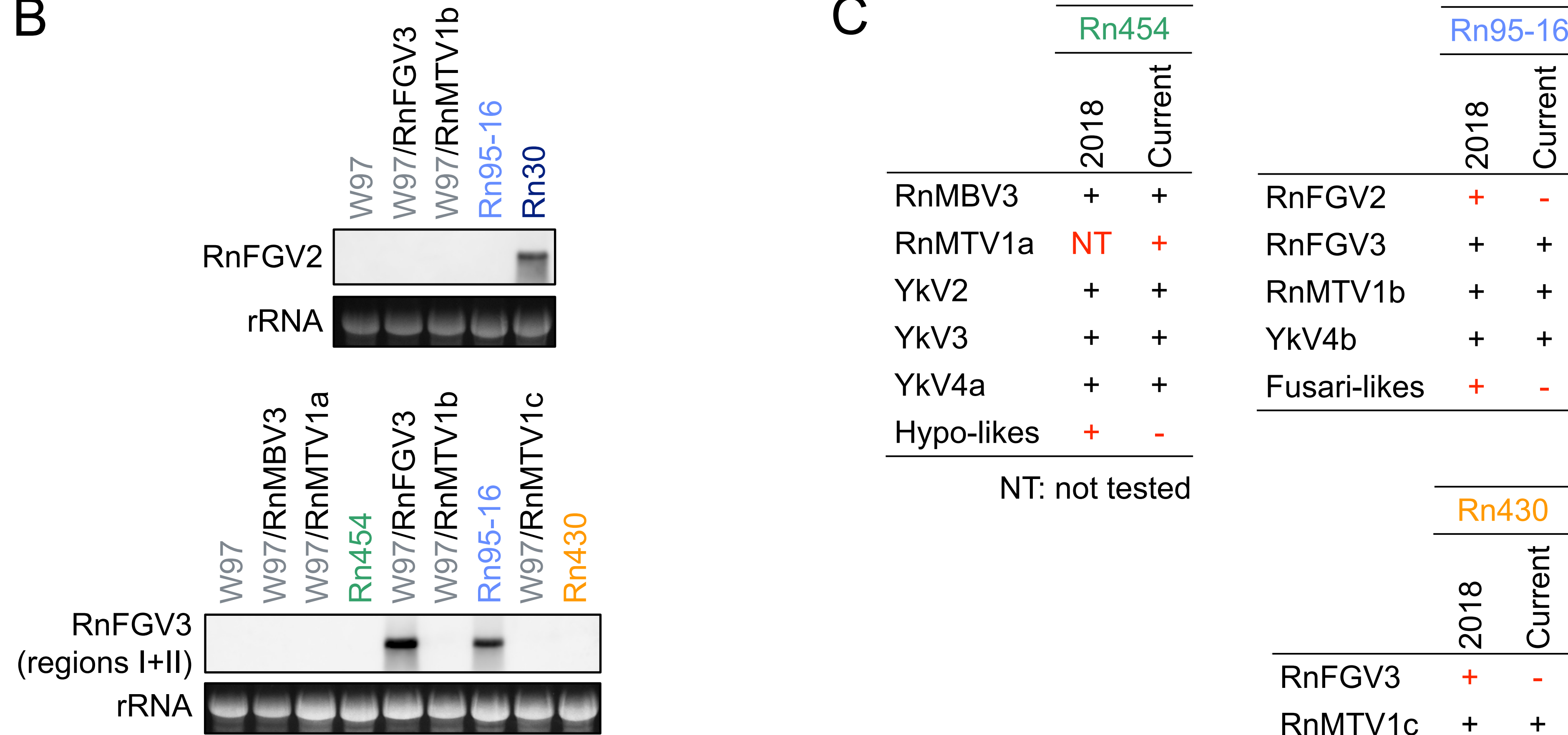

D

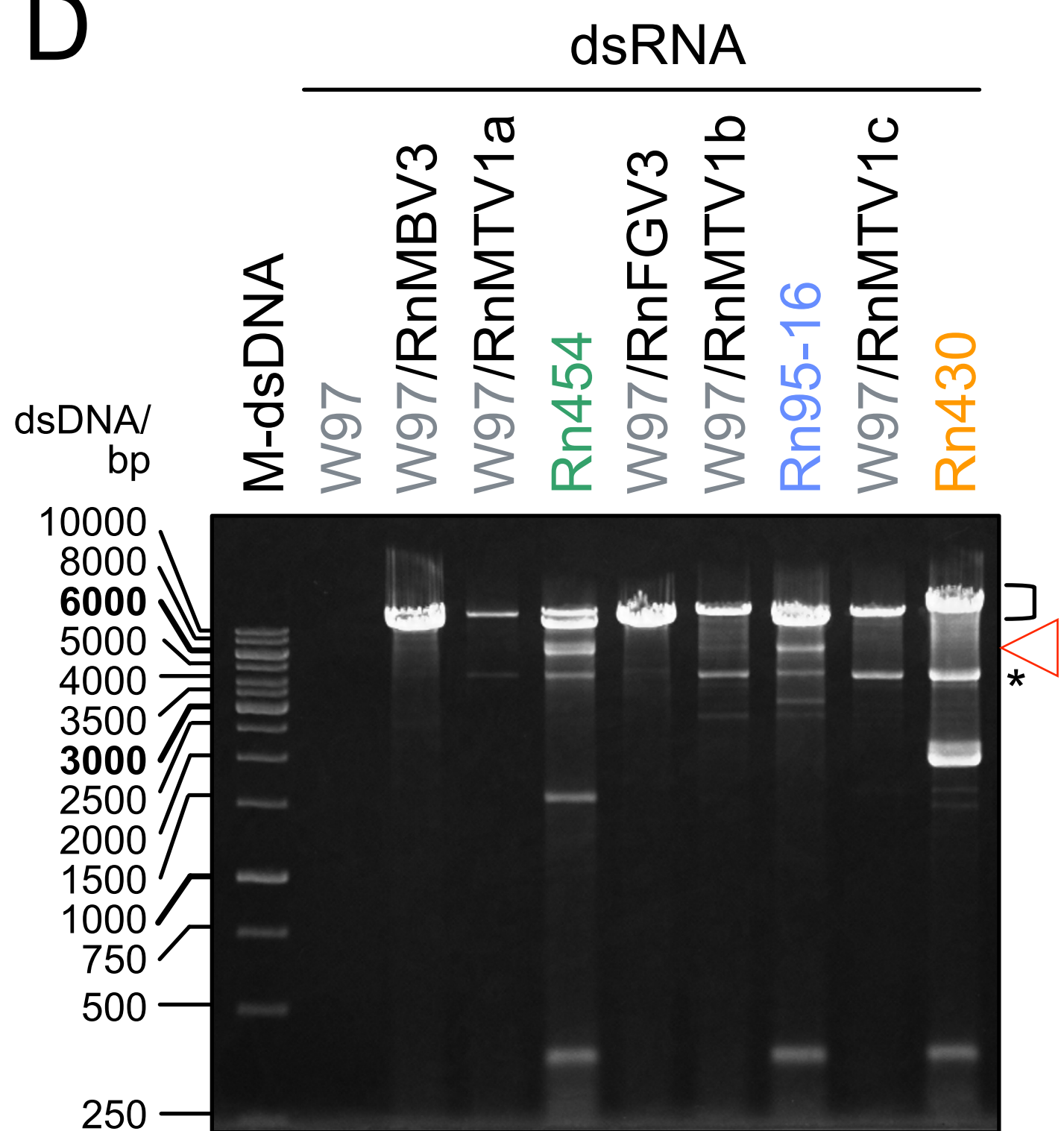

E

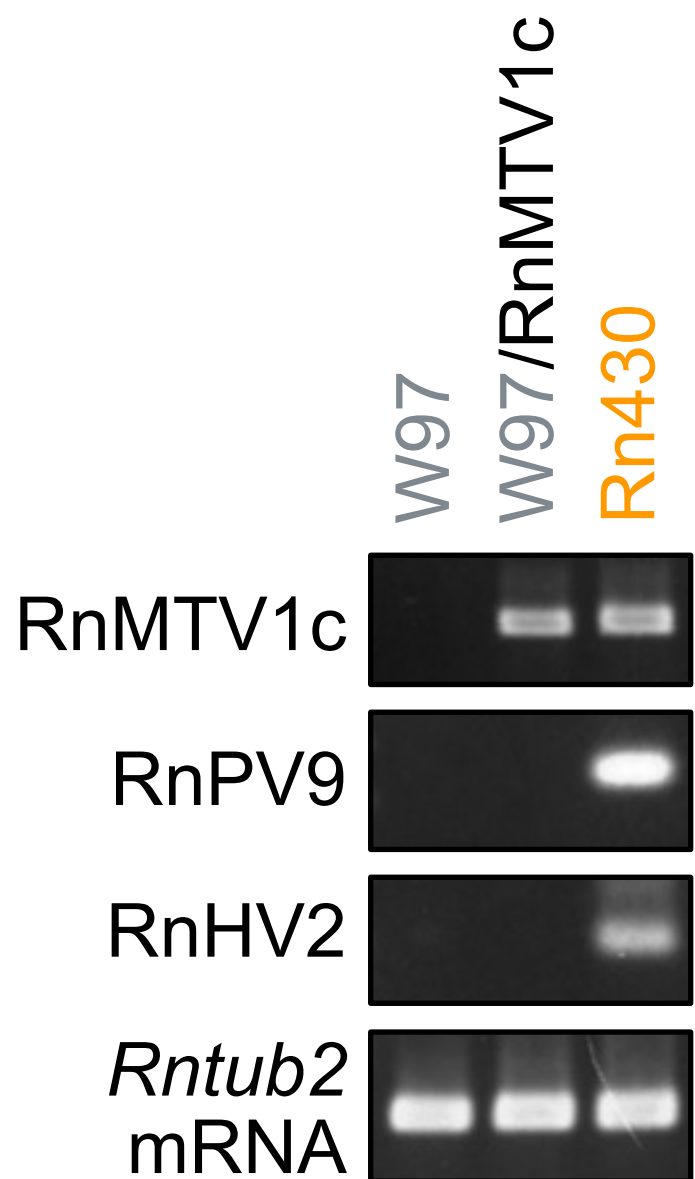

Supplement: FIG S2 [file mbio.01685-22-s0002.pdf]

Figure S3

A

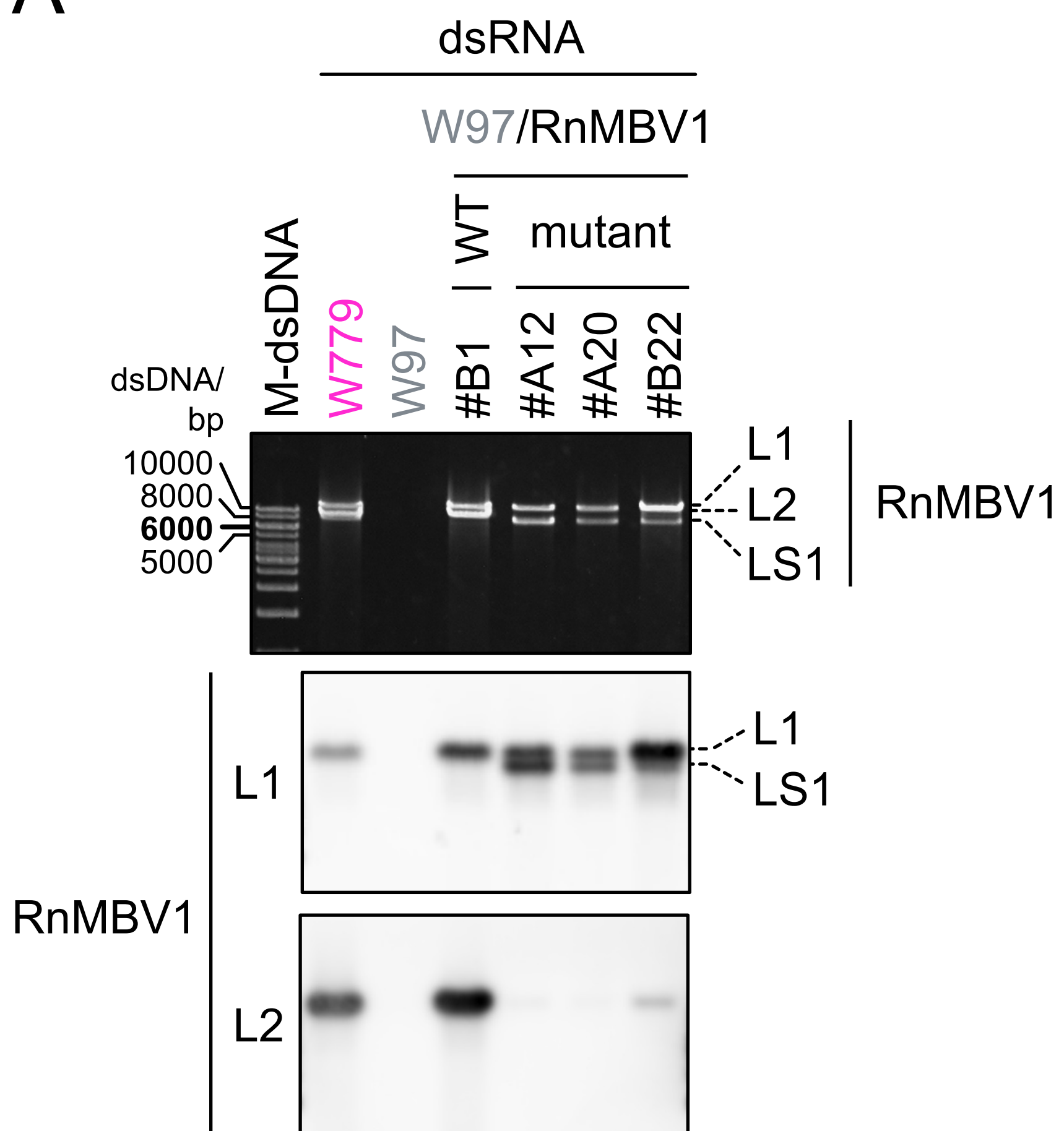

B

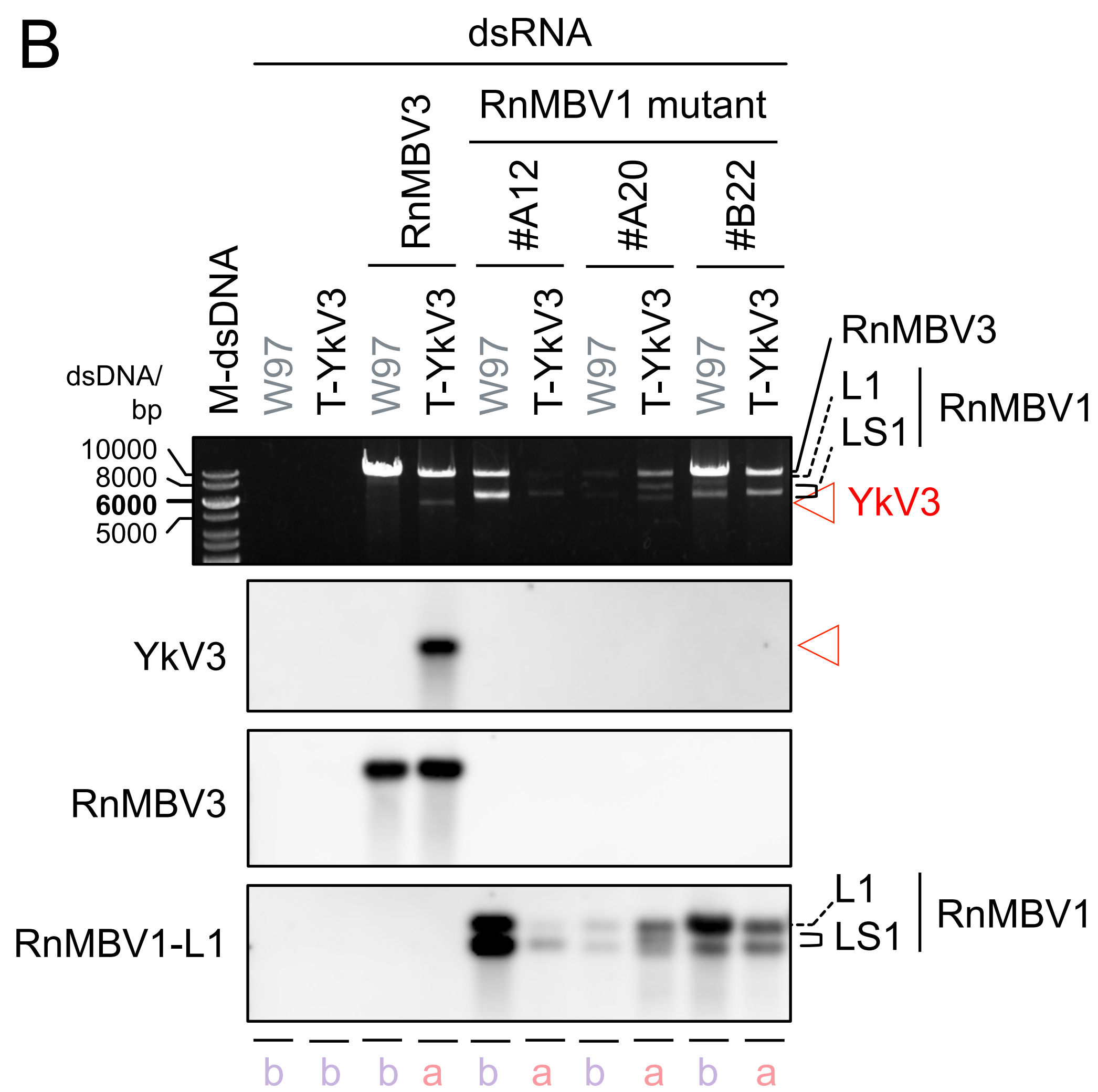

b: before co-culture

a: after co-culture with the W97 virus transfectant

Supplement: FIG S3 [file mbio.01685-22-s0003.pdf]

Figure S4

A

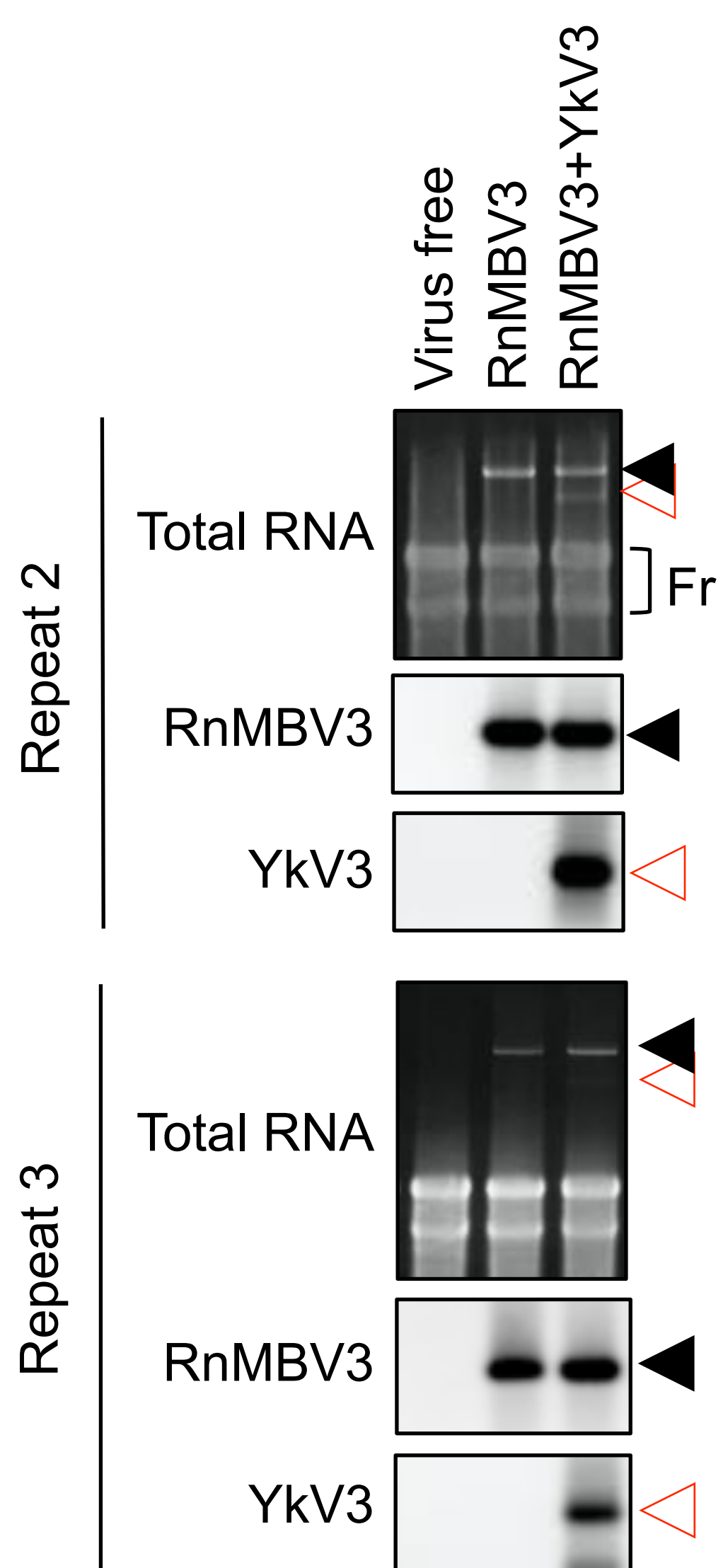

B

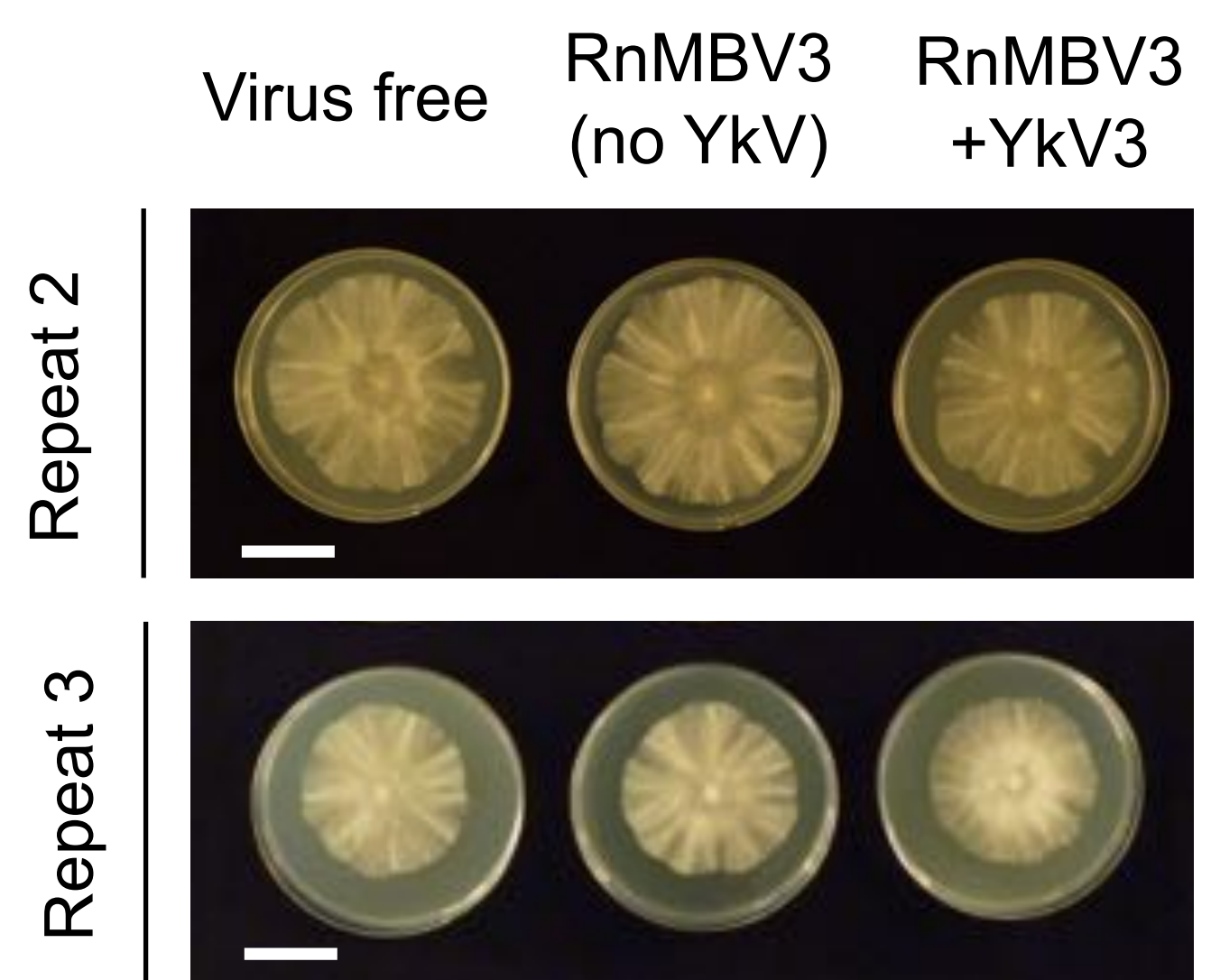

C

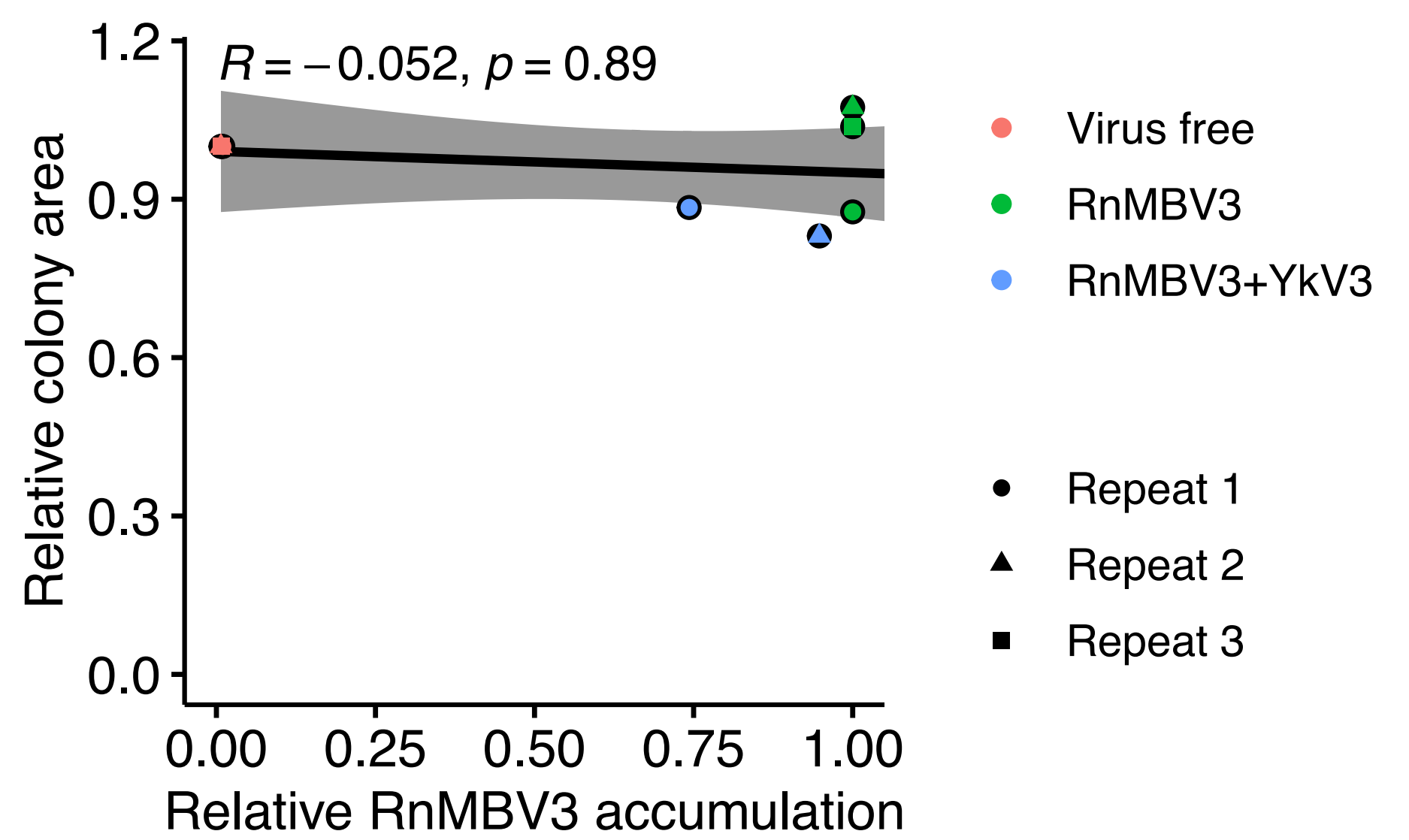

Supplement: FIG S4 [file mbio.01685-22-s0004.pdf]

Figure S5

A

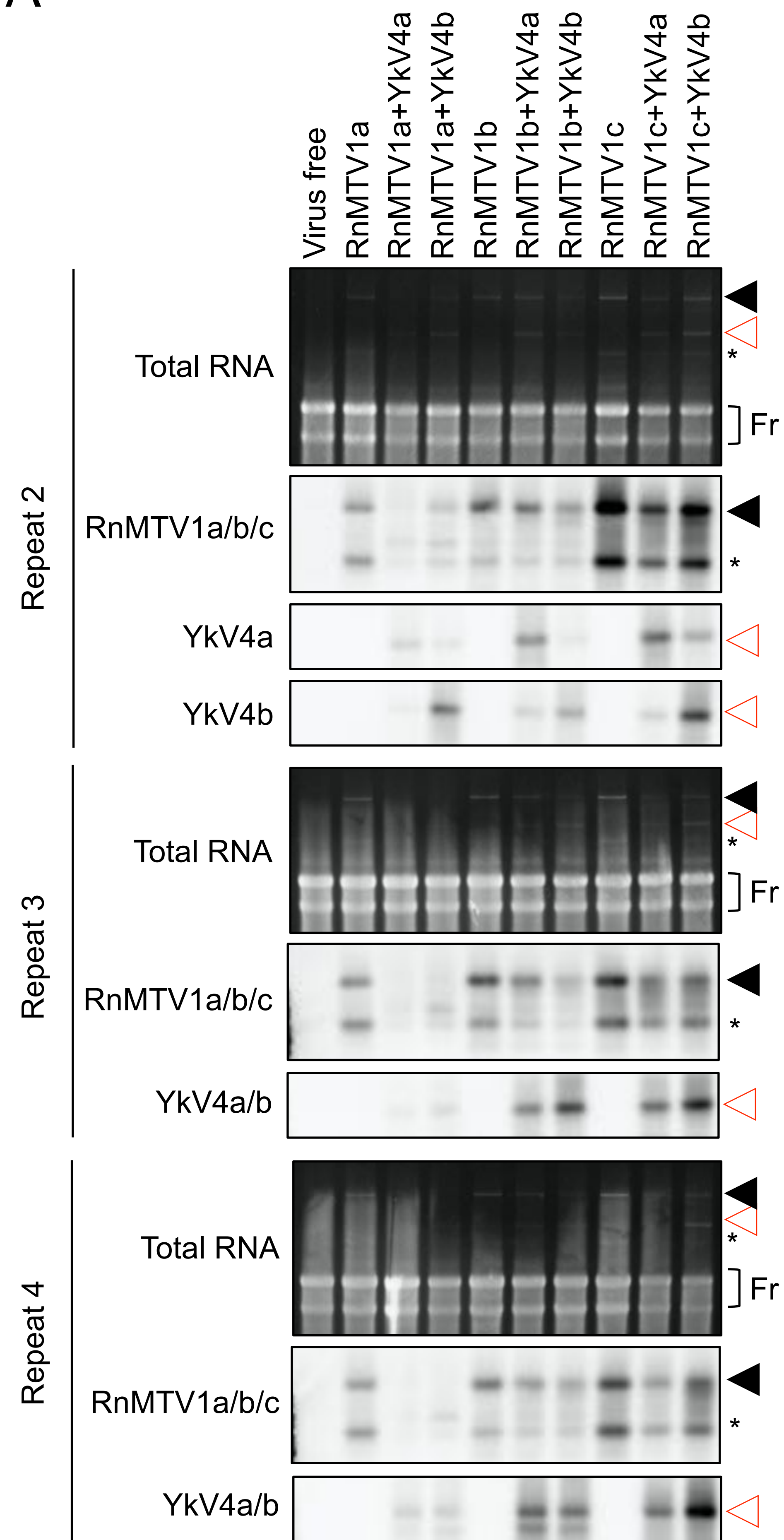

B

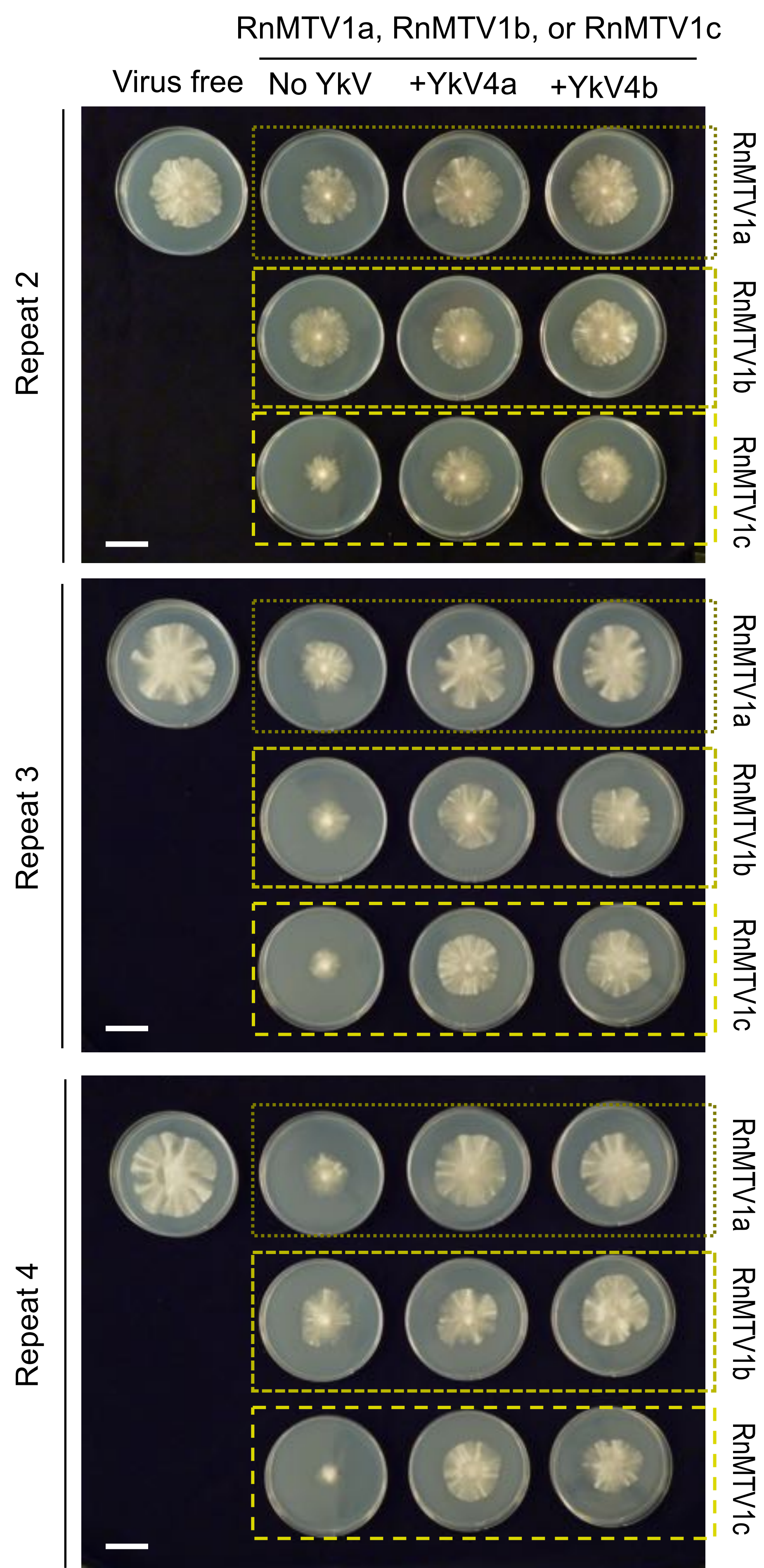

Supplement: FIG S5 [file mbio.01685-22-s0005.pdf]
