# Supplementary material for: Three-Layered Complex Interactions among Capsidless (+)ssRNA Yadokariviruses, dsRNA Viruses, and a Fungus
Source: mBio. 2022 Aug 30;13(5):e01685-22. doi: 10.1128/mbio.01685-22 (PMC9600902; doi:10.1128/mbio.01685-22)
Supplement: TABLE S1 [file mbio.01685-22-s0006.docx]

**Table S1. Viruses in the family *Yadokariviridae*.**

| **Genus** | **Species** | **Virus name** | **Virus abbrev.** | **RdRP accession** | **Reference** |
| --- | --- | --- | --- | --- | --- |
| *Alphayadokarivirus* | *Alphayadokarivirus ichibani* | Yado-kari virus 1 | YkV1 | [BAT50982.1](https://www.ncbi.nlm.nih.gov/protein/BAT50982.1) | (1) |
|  | Unassigned | Rhizoctonia solani mycovirus 1*^a^* | RsMV1 | [ANR02697.1](https://www.ncbi.nlm.nih.gov/protein/ANR02697.1?report=genbank&log$=prottop&blast_rank=9&RID=04FW5FA2013) | (4) |
|  | *Alphayadokarivirus nibani* | Aspergillus foetidus slow virus 2 | AfSV2 | [CCD33025.1](https://www.ncbi.nlm.nih.gov/protein/CCD33025.1?report=genbank&log$=prottop&blast_rank=5&RID=04FW5FA2013) | (5) |
|  |  | Aspergillus homomorphus yadokarivirus 1 *^b^* | AhoYV1 | [AZT88626.1](https://www.ncbi.nlm.nih.gov/protein/AZT88626.1?report=genbank&log$=prottop&blast_rank=3&RID=04FW5FA2013) | (6) |
|  | *Alphayadokarivirus sanbani* | Penicillium aurantiogriseum foetidus-like virus | PaFlV1 | [ALO50127.1](https://www.ncbi.nlm.nih.gov/protein/ALO50127.1) | (7) |
|  | *Alphayadokarivirus yonbani* | Penicillium digitatum yadokarivirus 1*^b^* | PdYV1 | [AZT88627.1](https://www.ncbi.nlm.nih.gov/protein/AZT88627.1?report=genbank&log$=prottop&blast_rank=7&RID=04FW5FA2013) | (6) |
|  | *Alphayadokarivirus gobani* | Plasmopara viticola lesion associated yadokari virus 1 | PvYV1 | [QHD64758.1](https://www.ncbi.nlm.nih.gov/protein/QHD64758.1?report=genbank&log$=prottop&blast_rank=8&RID=04FW5FA2013) | (8) |
|  | *Alphayadokarivirus rokubani* | Picoa juniperi yado-kari virus 1 | PjYV1 | [QOI17269.1](https://www.ncbi.nlm.nih.gov/protein/QOI17269.1?report=genbank&log$=prottop&blast_rank=11&RID=04FW5FA2013) | (9) |
| *Betayadokarivirus* | *Betayadokarivirus ichibani* | Fusarium poae mycovirus 2 | FpMyV2 | [BAV56311.1](https://www.ncbi.nlm.nih.gov/protein/YP_009272910.1?report=genbank&log$=prottop&blast_rank=12&RID=04FW5FA2013) | (10) |
|  | *Betayadokarivirus nibani* | Yado-kari virus 2/E97-14 | YkV2_/E97-14_ | [AVD68673.2](https://www.ncbi.nlm.nih.gov/protein/AVD68673.2?report=genbank&log$=prottop&blast_rank=14&RID=04FW5FA2013) | (11) |
|  |  | Yado-kari virus 2/Rn454 | YkV2 | [BBB86807.2](https://www.ncbi.nlm.nih.gov/protein/BBB86807.2?report=genbank&log$=prottop&blast_rank=15&RID=04FW5FA2013) | (1) |
|  | *Betayadokarivirus sanbani* | Yado-kari virus 3 | YkV3 | [BBB86810.2](https://www.ncbi.nlm.nih.gov/protein/BBB86810.2?report=genbank&log$=prottop&blast_rank=13&RID=04FW5FA2013) | (1) |
|  | *Betayadokarivirus yonbani* | Yado-kari virus 4/Rn454 | YkV4a | [BBB86805.1](https://www.ncbi.nlm.nih.gov/protein/BBB86805.1?report=genbank&log$=prottop&blast_rank=16&RID=04FW5FA2013) | (1) |
|  |  | Yado-kari virus 4/Rn95-16 | YkV4b | [BBB86788.1](https://www.ncbi.nlm.nih.gov/protein/BBB86788.1?report=genbank&log$=prottop&blast_rank=17&RID=04FW5FA2013) | (1) |
|  | Unassigned | Sclerotinia sclerotiorum yadokari virus 1 | SsYkV1 | UCR17164.1 | (12) |

*^a^*Partial sequence.

*^b^*Found from public fungal transcriptomic data.

1. Arjona-Lopez JM, Telengech P, Jamal A, Hisano S, Kondo H, Yelin MD, Arjona-Girona I, Kanematsu S, Lopez-Herrera CJ, Suzuki N. 2018. Novel, diverse RNA viruses from Mediterranean isolates of the phytopathogenic fungus, Rosellinia necatrix: insights into evolutionary biology of fungal viruses. Environ Microbiol 20:1464–1483.

4. Bartholomäus A, Wibberg D, Winkler A, Pühler A, Schlüter A, Varrelmann M. 2016. Deep Sequencing Analysis Reveals the Mycoviral Diversity of the Virome of an Avirulent Isolate of Rhizoctonia solani AG-2-2 IV. PLOS ONE 11:e0165965.

5. Kozlakidis Z, Herrero N, Ozkan S, Bhatti MF, Coutts RHA. 2013. A novel dsRNA element isolated from the Aspergillus foetidus mycovirus complex. Arch Virol 158:2625–2628.

6. Gilbert KB, Holcomb EE, Allscheid RL, Carrington JC. 2019. Hiding in plain sight: New virus genomes discovered via a systematic analysis of fungal public transcriptomes. PLOS ONE 14:e0219207.

7. Nerva L, Ciuffo M, Vallino M, Margaria P, Varese GC, Gnavi G, Turina M. 2016. Multiple approaches for the detection and characterization of viral and plasmid symbionts from a collection of marine fungi. Virus Res 219:22–38.

8. Chiapello M, Rodríguez-Romero J, Ayllón MA, Turina M. 2020. Analysis of the virome associated to grapevine downy mildew lesions reveals new mycovirus lineages. Virus Evolution 6:veaa058.

9. Sahin E, Keskin E, Akata I. 2021. Novel and diverse mycoviruses co-inhabiting the hypogeous ectomycorrhizal fungus Picoa juniperi. Virology 552:10–19.

10. Osaki H, Sasaki A, Nomiyama K, Tomioka K. 2016. Multiple virus infection in a single strain of Fusarium poae shown by deep sequencing. Virus Genes 52:835–847.

11. Velasco L, Arjona-Girona I, Cretazzo E, López-Herrera C. 2019. Viromes in Xylariaceae fungi infecting avocado in Spain. Virology 532:11–21.

12. Jia J, Mu F, Fu Y, Cheng J, Lin Y, Li B, Jiang D, Xie J. 2022. A Capsidless Virus Is trans-Encapsidated by a Bisegmented Botybirnavirus. Journal of Virology 96:e00296-22.
