# Supplementary material for: Three-Layered Complex Interactions among Capsidless (+)ssRNA Yadokariviruses, dsRNA Viruses, and a Fungus
Source: mBio. 2022 Aug 30;13(5):e01685-22. doi: 10.1128/mbio.01685-22 (PMC9600902; doi:10.1128/mbio.01685-22)
Supplement: TABLE S3 [file mbio.01685-22-s0008.docx]

**Table S3. Accession numbers for viral sequences used in this study.**

| **Virus** | **Virus**  **abbrev.** | **Accession** | | |
| --- | --- | --- | --- | --- |
|  |  | **Genome** | **CP** | **RdRP** |
| yado-kari virus 1/W1032 | YkV1 | [LC006253.1](https://www.ncbi.nlm.nih.gov/nuccore/LC006253) | - | [BAT50982.1](https://www.ncbi.nlm.nih.gov/protein/BAT50982.1) |
| yado-kari virus 2/Rn454 | YkV2 | [LC333755.2](https://www.ncbi.nlm.nih.gov/nuccore/LC333755) | - | BBB86807.2 |
| yado-kari virus 3/Rn454 | YkV3 | [LC333757.2](https://www.ncbi.nlm.nih.gov/nuccore/LC333757) | - | BBB86810.2 |
| yado-kari virus 4/Rn454 | YkV4a | [LC333754.2](https://www.ncbi.nlm.nih.gov/nuccore/LC333754) | - | BBB86805.1 |
| yado-kari virus 4/Rn95-16 | YkV4b | [LC333741.2](https://www.ncbi.nlm.nih.gov/nuccore/LC333741) | - | BBB86788.1 |
| yado-nushi virus 1-A/W1032 | YnV1-A | [LC061478.1](https://www.ncbi.nlm.nih.gov/nuccore/LC061478) | [BAT50986.1](https://www.ncbi.nlm.nih.gov/protein/952545738) | [BAT50987.1](https://www.ncbi.nlm.nih.gov/protein/952545739) |
| Rosellinia necatrix megabirnavirus 1/  W779 | RnMBV1 | [AB512282.1](https://www.ncbi.nlm.nih.gov/nuccore/AB512282), [AB512283.1](https://www.ncbi.nlm.nih.gov/nuccore/AB512283) | [BAI48015.1](https://www.ncbi.nlm.nih.gov/protein/BAI48015.1) | [BAI48016.1](https://www.ncbi.nlm.nih.gov/protein/BAI48016.1) |
| Rosellinia necatrix megabirnavirus 3/  Rn454 | RnMBV3 | [LC333756.2](https://www.ncbi.nlm.nih.gov/nuccore/LC333756) | [BBB86808.1](https://www.ncbi.nlm.nih.gov/protein/1325803813) | [BBB86809.1](https://www.ncbi.nlm.nih.gov/protein/1325803814) |
| Rosellinia necatrix megatotivirus 1/  Rn454 | RnMTV1a | LC650957.1 | [BDB32682.1](https://www.ncbi.nlm.nih.gov/protein/BDB32682.1) | [BDB32683.1](https://www.ncbi.nlm.nih.gov/protein/BDB32683.1) |
| Rosellinia necatrix megatotivirus 1/  Rn95-16 | RnMTV1b | [LC333740.3](https://www.ncbi.nlm.nih.gov/nuccore/LC333740.3) | [BBB86786.2](https://www.ncbi.nlm.nih.gov/protein/2131580516) | [BBB86787.1](https://www.ncbi.nlm.nih.gov/protein/1325803776) |
| Rosellinia necatrix megatotivirus 1/  Rn430 | RnMTV1c | [LC333746.2](https://www.ncbi.nlm.nih.gov/nuccore/LC333746) | [BBB86795.1](https://www.ncbi.nlm.nih.gov/protein/1325803790) | [BBB86796.1](https://www.ncbi.nlm.nih.gov/protein/1325803791) |
| Rosellinia necatrix fusagravirus 3/  Rn95-16 | RnFGV3 | [LC333739.2](https://www.ncbi.nlm.nih.gov/nuccore/LC333739) | [BBB86784.1](https://www.ncbi.nlm.nih.gov/protein/1325803772) | [BBB86785.2](https://www.ncbi.nlm.nih.gov/protein/1339015200) |
