# Supplementary material for: Three-Layered Complex Interactions among Capsidless (+)ssRNA Yadokariviruses, dsRNA Viruses, and a Fungus
Source: mBio. 2022 Aug 30;13(5):e01685-22. doi: 10.1128/mbio.01685-22 (PMC9600902; doi:10.1128/mbio.01685-22)
Supplement: TABLE S2 [file mbio.01685-22-s0007.docx]

**Table S2. Viruses in the order *Ghabrivirales* used for phylogenetic analysis.**

| **Sub-order** | **Family** | **Genus** | **Virus** | **RdRP Accession** |
| --- | --- | --- | --- | --- |
| Alphatotivirineae | Unclassified | *Botybirnavirus* | Sclerotinia sclerotiorum botybirnavirus 1 | YP_009141011.1 |
|  |  |  | Botrytis porri botybirnavirus 1 | AFD04798.1 |
|  |  |  | Plasmopara viticola lesion associated botybirna 1 | QGZ98417.1 |
|  |  |  | Sclerotinia sclerotiorum botybirnavirus 3 | UOJ41050.1 |
|  | *Megabirnaviridae* | *Megabirnavirus* | Rosellinia necatrix megabirnavirus 1 | BAI48016.1 |
|  | Unclassified (*Megabirnaviridae*) | Unclassified (*Megabirnavirus*) | Sclerotinia sclerotiorum megabirnavirus 1 | YP_009143529.1 |
|  |  |  | Rosellinia necatrix megabirnavirus 3 | BBB86809.1 |
|  |  |  | Entoleuca megabirnavirus 1 | AVD68671.1 |
|  | *Chrysoviridae* | *Alphachrysovirus* | Penicillium chrysogenum virus | AAM95601.1 |
|  |  |  | Shuangao chryso-like virus 1 | ASA47445.1 |
|  |  | *Betachrysovirus* | Botryosphaeria dothidea chrysovirus 1 | AGZ84312.1 |
|  |  |  | Fusarium oxysporum f. sp. dianthi mycovirus 1 | AKP45145.1 |
|  | Fusagraviridae | - | Rosellinia necatrix fusagravirus 3 | BBB86785.2 |
|  |  |  | Rosellinia necatrix fusagravirus 2 | BBB86783.1 |
|  |  |  | Phlebiopsis gigantea mycovirus dsRNA 2 | CAJ34335.2 |
|  |  |  | Fusarium poae mycovirus 1 | YP_009272909.1 |
|  |  |  | Rosellinia necatrix fusagravirus 1/Rn_Wito-3-CH | BBB86778.1 |
|  |  |  | Rosellinia necatrix fusagravirus 1/E97-14 | AZG06258.1 |
|  |  |  | Picoa juniperi mycovirus 1 | QOI17266.1 |
|  |  |  | Picoa juniperi mycovirus 2 | QOI17268.1 |
|  | *Quadriviridae* | *Quadrivirus* | Rosellinia necatrix quadrivirus 1 | BAL46425.1 |
|  | Unclassified Quadri-like | Unclassified Quadri-like | Amasya cherry disease-associated mycovirus | CAJ29958.1 |
|  | *Totiviridae* | *Totivirus* | Saccharomyces cerevisiae virus L-A | AAA50508.1 |
|  |  |  | Tuber aestivum virus 1 | YP_009507833.1 |
|  |  | *Victorivirus* | Aspergillus homomorphus totivirus 1 | AZT88629.1 |
|  |  |  | Penicillium aurantiogriseum totivirus 1 | ALN98256.1 |
|  |  |  | Coniothyrium minitans RNA virus | AAO14999.1 |
|  |  |  | Fusarium poae victorivirus 1 | BAV56302.1 |
|  |  |  | Helminthosporium victoriae virus 190S | AAB94791.2 |
|  |  |  | Aspergillus foetidus slow virus 1 | CCD33024.1 |
|  |  | *Leishmaniavirus* | Leishmania RNA virus 1 - 1 | AAB50024.1 |
|  |  |  | Leishmania RNA virus 2 - 1 | AAB50031.1 |
|  |  | *Trichomonasvirus* | Trichomonas vaginalis virus 1 | AAA62868.1 |
|  |  |  | Trichomonas vaginalis virus 2 | AAF29445.1 |
| Betatotivirineae | *Totiviridae* | *Giardiavirus* | Giardia lamblia virus | AAB01579.1 |
|  | Yadonushiviridae | - | Yado-nushi virus 1-A | YP_009551446.1 |
|  |  |  | Yado-nushi virus 1-B | BAU20287.1 |
|  |  |  | Yado-nushi virus 1-C | BAU20289.1 |
|  |  |  | Ceratobasidium virus 1/Murdoch-2 | AOX47544.1 |
|  |  |  | Ceratobasidium virus 1/Murdoch-4 | AOX47585.1 |
|  | Megatotiviridae | - | Rosellinia necatrix megatotivirus 1/Rn454 | BDB32683.1 |
|  |  |  | Rosellinia necatrix megatotivirus 1/Rn95-16 | BBB86787.1 |
|  |  |  | Rosellinia necatrix megatotivirus 1/Rn430 | BBB86796.1 |
|  |  |  | Picoa juniperi megatotivirus 1 | QOI17264.1 |
