# Supplementary material for: Three-Layered Complex Interactions among Capsidless (+)ssRNA Yadokariviruses, dsRNA Viruses, and a Fungus
Source: mBio. 2022 Aug 30;13(5):e01685-22. doi: 10.1128/mbio.01685-22 (PMC9600902; doi:10.1128/mbio.01685-22)
Supplement: TABLE S4 [file mbio.01685-22-s0009.docx]

**Table S4. Primers used for cloning of the infectious cDNA clone of YkV4b.**

| **For** | **Target** | **Primer sequence (5'-3')** |
| --- | --- | --- |
| Sub-cloning | 5’-half of YkV4b | CGGGGGTAGCCCACACCGAAG |
|  |  | CAGCCAGTTTTTCTTTTTCTT |
|  | 3’-half of YkV4b | TCCATGGTATACAATTACAAA |
|  |  | TTTTTTTTTTCGACGTTGTCG |
| In-Fusion cloning *^a^* | 5’-half of YkV4b | CTTGTTAACGCGGCCCGGGGGTAGCCCACACCGAAGTAT |
|  |  | TTCTTTTTCTTGCATCTTTTCCGTCTCGAA |
|  | 3’-half of YkV4b | ATGCAAGAAAAAGAAAAACTGGCTGTTAAA |
|  |  | AAGCATGCGCGGCCGCTTTTTTTTTTTTTTTTTTTTCGAC |

*^a^* The underlined sequences were attached for In-Fusion cloning into pCPXHY3 linearized by *Not* I.
