# Supplementary material for: Three-Layered Complex Interactions among Capsidless (+)ssRNA Yadokariviruses, dsRNA Viruses, and a Fungus
Source: mBio. 2022 Aug 30;13(5):e01685-22. doi: 10.1128/mbio.01685-22 (PMC9600902; doi:10.1128/mbio.01685-22)
Supplement: TABLE S5 [file mbio.01685-22-s0010.docx]

**Table S5. Primers used for RT-PCR, DIG-labelling PCR, or genotyping.**

| **Target** | **Primer sequence (5'-3')** | **Expected size (bp)** | **Reference** |
| --- | --- | --- | --- |
| YkV1 | CGGTCCTCCGATCGAAGATG | 379 | This study |
|  | TGAGGCCTTAGGGTGATCCA |  |  |
| YkV2 | ACGAAGCCGATGCACTAACAC | 510 | (1) |
|  | CCATGTGGAACGCCGGTAAAT |  |  |
| YkV3 | GAAGAATCTCAACCATGGAGA | 500 |  |
|  | GGGCAGCCCCCACTATCATAC |  |  |
| YkV4a | CGCACTCTCTTCAATCGAGCA | 520 |  |
|  | TATCCGGGAATATTTATCGAG |  |  |
| YkV4b | AGTGGAATACCCTTTACAAAA | 500 |  |
|  | CAAGAAACTGTCATAGGTAAA |  |  |
| YnV1-A | GTCAGGACTACGCCATCGAG | 501 | This study |
|  | GAGCGATACCAAGTCCTCCG |  |  |
| RnMBV1-L1 | GGCCATGAGGCTGTTGTAGT | 482 | This study |
|  | GCTGCAGTGTGGATCTCCTT |  |  |
| RnMBV1-L2 | GCGCTAGCCAATGTGGAATG | 702 | This study |
|  | CATGGCATCCAAATCCTGCG |  |  |
| RnMBV3 | TTCGGGCAGTGCAGGCAAGGG | 500 | (1) |
|  | GCGCTCGCACACCAGTTGGCC |  |  |
| RnMTV1a/b/c*^a^* | AATGACCCATCTAAGTGGGCA | 1043 | This study |
|  | AGGATGTTGTCGACATCTGAG |  |  |
| RnMTV1b*^b^* | CGGCCAACGTATTACTAATGG | 510 | (1) |
|  | ACGTTGTAGTGTTTTAGGATG |  |  |
| RnMTV1c*^b^* | TATTGGGTCACTTTCCTTAAG | 510 |  |
|  | GGCCGATAAAACATATCAAGT |  |  |
| RnFGV2 | ATCACACTTTCGATGCGTATT | 500 |  |
|  | TAAACCAGTCTCGCCGTCGTA |  |  |
| RnFGV3 (region I) | GGGTGTCCGTGTAGTAAAGTA | 500 |  |
|  | CTCCCTTCAAAGCGGTCAACG |  |  |
| RnFGV3 (region II) | GAAAGCAGTTGGAGAGAACGC | 750 | This study |
|  | AGCGTTAGAAACGGTAGGGGG |  |  |
| Hypo-like (contig 3-16) | GACCTCGTGTAATTGTCCTTT | 500 | (1) |
|  | TGATCTTACCGTACATCGTTC |  |  |
| Hypo-like (contig 3-41) | ATCATCATCCCCCGTATTATA | 500 |  |
|  | GATGTGTACAACTCAGACCCG |  |  |
| Hypo-like (contig 3-42) | CCCGTATTGTACAAAGGGTGG | 510 |  |
|  | GACGTGTACAACTCAGACCCG |  |  |
| Hypo-like (contig 3-388) | AACACAGTTCCGAAAGGTGCC | 500 |  |
|  | AGATACCTTCCATTACCTCGA |  |  |
| Fusari-like (contig 2-405) | AGTTGGGATTATTATTGATGA | 510 |  |
|  | ATAAAAGAATTGAAGGGTCAC |  |  |
| Fusari-like (contig 2-1109) | GTGTGAAAGGGATCGCGCGAA | 500 |  |
|  | ATGGGTGTCCTCCGGACTTGA |  |  |
| Fusari-like (contig 2-1110) | TAGAAACTCTCTCCCTTGTCC | 350 |  |
|  | CATGGAGATGACTTTAGTGGT |  |  |
| *Rntub2* | GGTAACCAAATCGGTGCTGCTTTC | 421 (genome),  ≈290 (mRNA) | (13) |
|  | ACCCTCAGTGTAGTGACCCTTGGC |  |  |

*^a^*The primers were used for the detection of RnMTV1a in Figs. 2A and 6B and of RnMTV1a/b/c in Fig. S5A.

*^b^*The primers were used for the detection of RnMTV1b or RnMTV1c in Figs. 2A, S2E, 6C and D.

1. Arjona-Lopez JM, Telengech P, Jamal A, Hisano S, Kondo H, Yelin MD, Arjona-Girona I, Kanematsu S, Lopez-Herrera CJ, Suzuki N. 2018. Novel, diverse RNA viruses from Mediterranean isolates of the phytopathogenic fungus, Rosellinia necatrix: insights into evolutionary biology of fungal viruses. Environ Microbiol 20:1464–1483.

13. Glass NL, Donaldson GC. 1995. Development of primer sets designed for use with the PCR to amplify conserved genes from filamentous ascomycetes. Appl Environ Microbiol 61:1323–1330.
